# Strength Degradation of Sandstone Under Coupled Loading and Freeze–Thaw Cycles: Experimental Study and Discrete Element Numerical Simulation

**DOI:** 10.3390/ma19163483

**Published:** 2026-08-18

**Authors:** Yingxiang Sun, Yuxin Bai, Jun Hou, Mingjie Chen, Lingren Meng, Penghai Zhang

**Affiliations:** 1 Zhaojin International Gold Co., Ltd., Jinan 250014, China; yingxiang.s@vgml.com.fj; 2 School of Resources and Civil Engineering, Northeastern University, Shenyang 110819, China; 3 Changchun Gold Research Institute Co., Ltd., Changchun 130012, China

**Keywords:** load–freeze–thaw coupling, sandstone, strength degradation, frost-heave damage, discrete element method

## Abstract

Previous studies have mainly examined sandstone freeze–thaw degradation under unloaded conditions, whereas the continuous influence of sustained stress on compressive and tensile properties remains unclear. To address this issue, water-saturated specimens were subjected to axial loads of 0–2 MPa and 0–10 freeze–thaw cycles, followed by uniaxial compression and Brazilian splitting tests. A two-variable exponential strength model and a load–freeze–thaw coupled discrete element model were established. Both strengths decreased with increasing freeze–thaw cycles. Under no load, after 10 cycles, the uniaxial compressive strength and tensile strength decreased by 27.85% and 36.67%, respectively, indicating higher freeze–thaw sensitivity of the tensile property. Loading mitigated strength loss: after 10 cycles, the two strengths of the 2 MPa group were 9.59% and 10.53% higher than those of the no-load group. The retention effect on compressive strength first increased and then decreased, whereas that on tensile strength increased with cycle number. The discrete element results showed that frost-heave cracks evolved from local initiation to connection and clustering, with tensile cracks dominating. Loading reduced tensile bond breakage and slowed crack accumulation and subsequent coalescence, linking the observed strength retention to the inhibition of mesoscopic tensile damage.

## 1. Introduction

Rock masses in cold-region slopes, tunnels, and underground engineering are subjected to the combined effects of low temperature, moisture, and stress over long periods. Frost heaves, induced by the phase transition of pore water, can alter the pore structure of rock and induce crack propagation. Previous studies have shown that as the number of freeze–thaw cycles increases, the P-wave velocity, uniaxial compressive strength, and elastic modulus of sandstone generally decrease, whereas the porosity and irreversible deformation gradually increase [1,2,3,4]. Song et al. [5] revealed the mesoscopic fracture evolution of sandstone under freeze–thaw cycling using computed tomography. In the early stage of freeze–thaw cycling, the damage was mainly manifested as the expansion of primary pores. With damage accumulation, adjacent pores and microcracks gradually connected, eventually affecting the macroscopic failure path. Compared with the compressive response, studies on the tensile properties of sandstone after freeze–thaw treatment remain relatively limited. Zhang et al. [6] found that freeze–thaw cycles also reduced the static and dynamic tensile strengths and changed crack propagation and specimen fragmentation characteristics. Existing studies have provided a relatively systematic understanding of the degradation of macroscopic mechanical properties and mesoscopic structures of sandstone after freeze–thaw treatment without load, but they cannot adequately reflect the continuous influence of stress on fracture states and frost-heave damage during freeze–thaw cycling.

Rock masses in engineering practice are usually subjected to self-weight, in situ stress, or structural loads during thermal cycling, and their freeze–thaw damage does not occur under completely free boundary conditions. Liu et al. [7] investigated pore-structure changes in water-rich sandstone induced by freeze–thaw processes under different confining pressures using digital rock technology. Liu et al. [8] further examined the mechanical and hydraulic properties of sandstone under freeze–thaw cycling and showed that freeze–thaw cycling affected its mechanical response and permeability evolution. Loaded specimens may maintain relatively high bearing capacity during the early stage of freeze–thaw cycling, whereas higher loads may aggravate mechanical degradation after prolonged cycling [9]. Static and dynamic loading tests after freeze–thaw treatment have also confirmed that the loading mode and strain rate can change the strength and fragmentation degree of damaged sandstone [10]. Similar coupled effects have also been reported in concrete. Chen et al. [11] investigated the durability and bearing capacity of reinforced concrete columns under sustained loading and freeze–thaw cycles, while Li et al. [12] examined the flexural performance of reinforced geopolymer concrete beams subjected to freeze–thaw cycles and sustained loading. These studies further demonstrate that external loading can influence the freeze–thaw deterioration of porous brittle materials. The existing studies on load–freeze–thaw coupling in sandstone mainly focus on deformation, compressive strength, and permeability, while systematic comparisons of the simultaneous changes in compressive and tensile properties under loading remain limited. The controlling effect of loading on the frost-heave-induced degradation of these two strength indices remains unclear.

The discrete element method can describe the initiation, propagation, and coalescence of rock cracks from the perspective of particle contacts and bond breakage, providing an effective approach for linking macroscopic strength degradation with mesoscopic damage evolution. Existing numerical methods for freeze–thaw damage can generally be divided into two categories. One category equivalently introduces freeze–thaw damage by reducing the contact stiffness, bond strength, or macroscopic mechanical parameters [13]. The other treats pore water or ice as an independent medium and explicitly reproduces the generation of frost-heave cracks through particle expansion, contact interaction, and bond breakage [14,15]. On this basis, related studies have further introduced CT-derived pore structures, heat transfer processes, and bond-expansion coupling relationships to analyze the strength degradation and mesoscopic crack accumulation during freeze-thaw cycling [16,17]. These studies have promoted the development of freeze–thaw simulation from empirical parameter reduction toward the explicit representation of frost-heave processes. However, current simulations are still mainly conducted under unloaded free-boundary freeze–thaw conditions, and few studies have used load–freeze–thaw coupled simulations to analyze the mechanism by which loading affects mesoscopic damage.

To address these issues, water-saturated sandstone specimens were first subjected to load–freeze–thaw coupled pretreatment, and the degradation patterns of compressive and tensile strengths were compared through uniaxial compression and Brazilian splitting tests. Based on the experimental results, a load–freeze–thaw coupled discrete element model was established using PFC 5.0. The damage accumulation process was simulated through temperature transfer, frost heave of equivalent water particles, and bond breakage, and the effects of the load–freeze–thaw coupling on crack failure types and failure modes during subsequent loading were analyzed. The main contribution of this study is the combined comparison of compressive and tensile strength degradation under sustained loading and the linkage of macroscopic strength retention with mesoscopic crack-type evolution through a load–freeze–thaw coupled DEM model.

## 2. Materials and Methods

### 2.1. Sandstone Specimens

The sandstone used in the tests was generally brown, with medium compactness, a small number of natural microcracks inside, and no obvious surface weathering. According to the relevant requirements of the International Society for Rock Mechanics (ISRM), the sandstone was processed into cylindrical specimens with a diameter of 50 mm and a height of 100 mm. The deviations in specimen diameter and height were both within ±0.3 mm, and the end-face flatness deviation was within ±0.5 mm.

**Figure 1:**
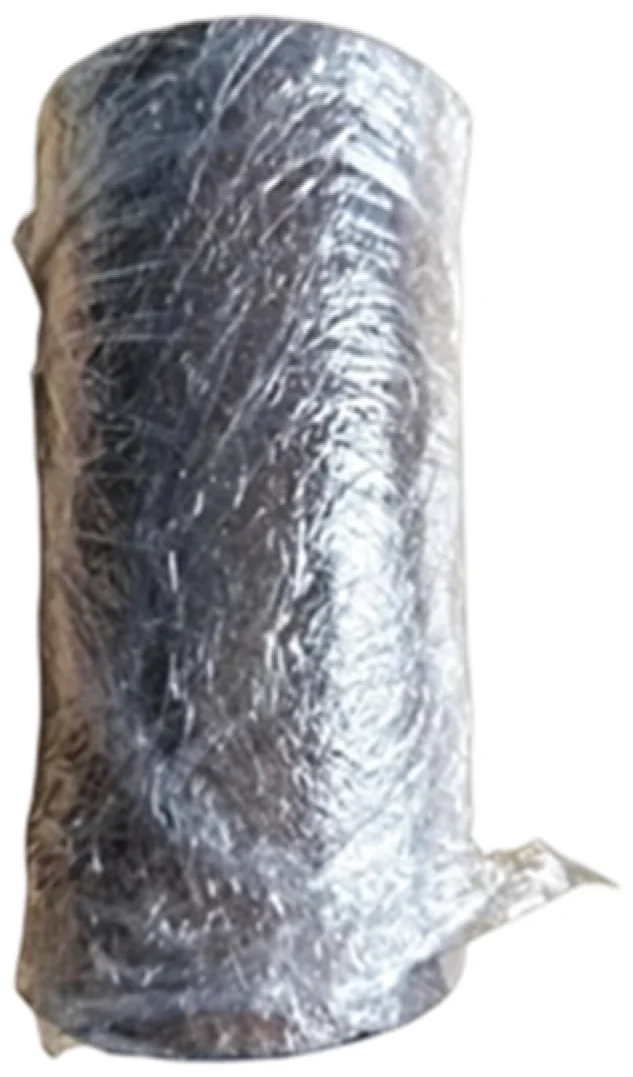
Sealed water-saturated sandstone specimen.

The selected specimens were completely immersed in distilled water and soaked for 120 h. When the specimen mass remained stable for 24 h, the specimens were considered to have reached a water-saturated state. The free water on the specimen surface was then wiped off, and the specimens were sealed with plastic bags and 3–4 layers of cling film to reduce water loss during freeze–thaw cycling, as shown in Figure 1.

### 2.2. Load–Freeze–Thaw Coupling Scheme

**Figure 2:**
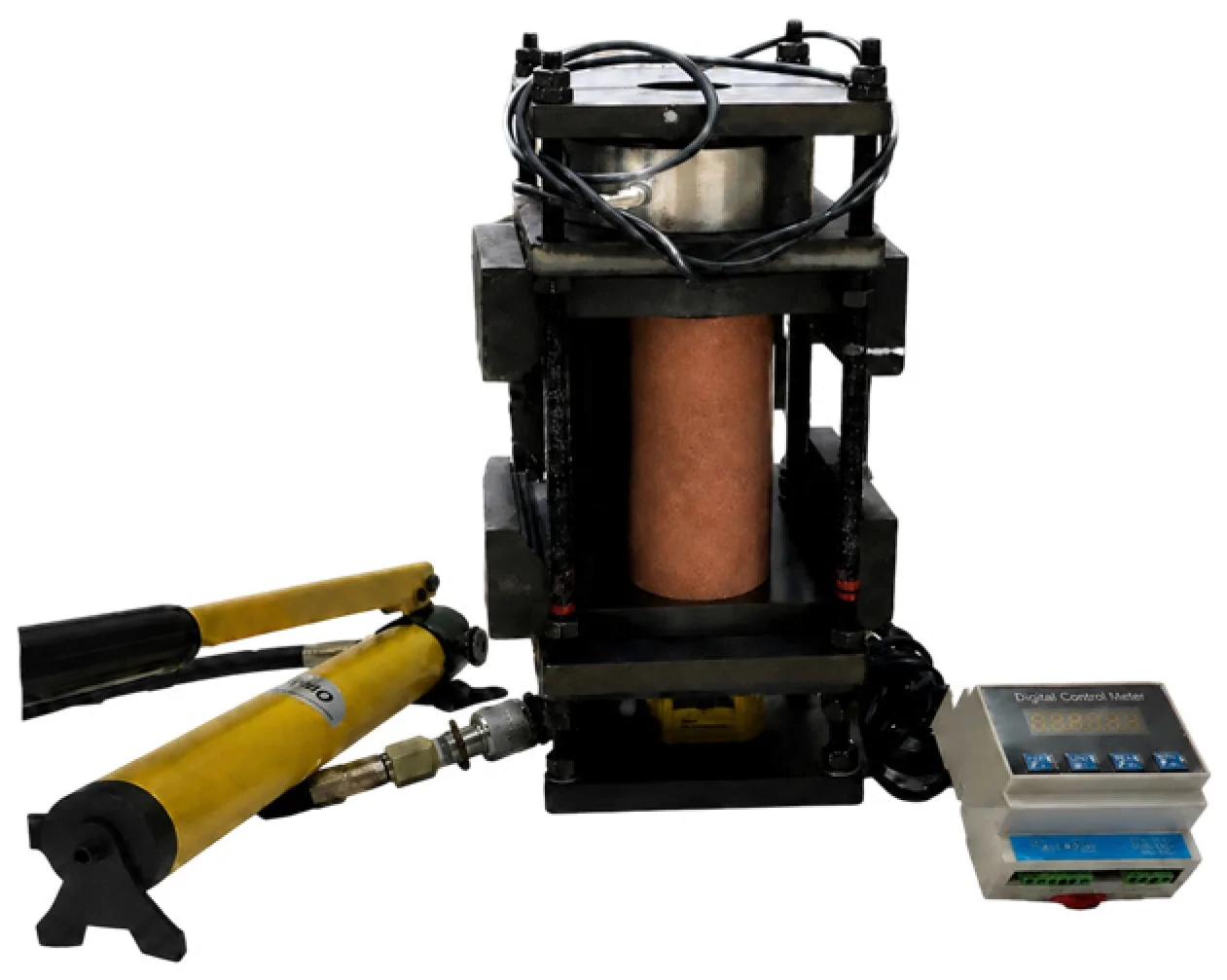
Compact apparatus for applying sustained axial loading.

A compact sustained loading apparatus was used to apply axial compressive stress to the specimens, and the loading apparatus and specimens were placed together in the freeze–thaw chamber. As shown in Figure 2, the load was applied by a hydraulic jack, and the load variation was monitored in real time using a pressure sensor. During the test, the load fluctuation was controlled within ±0.05 kN. For cylindrical specimens with a diameter of 50 mm, axial stresses of 1 MPa and 2 MPa corresponded to axial loads of approximately 1.96 kN and 3.93 kN, respectively.

The number of freeze–thaw cycles was set to 0, 1, 3, 5, and 10, and the sustained axial stress was set to 0, 1, and 2 MPa. The 0 MPa group was used as the single freeze–thaw control group, whereas the 1 MPa and 2 MPa groups were used as load–freeze–thaw coupled groups. The axial stresses of 1 MPa and 2 MPa were approximately 1.8% and 3.6% of the initial uniaxial compressive strength of sandstone, respectively. These relatively low stress levels were selected to investigate the influence of sustained loading on freeze–thaw damage while avoiding additional mechanical damage directly induced by a high sustained load. The investigated range of 0–2 MPa, corresponding to an equivalent burial range of approximately 0–80 m, covers the common shallow depth range in which self-weight stress and seasonal freeze–thaw action may coexist.

**Figure 3:**
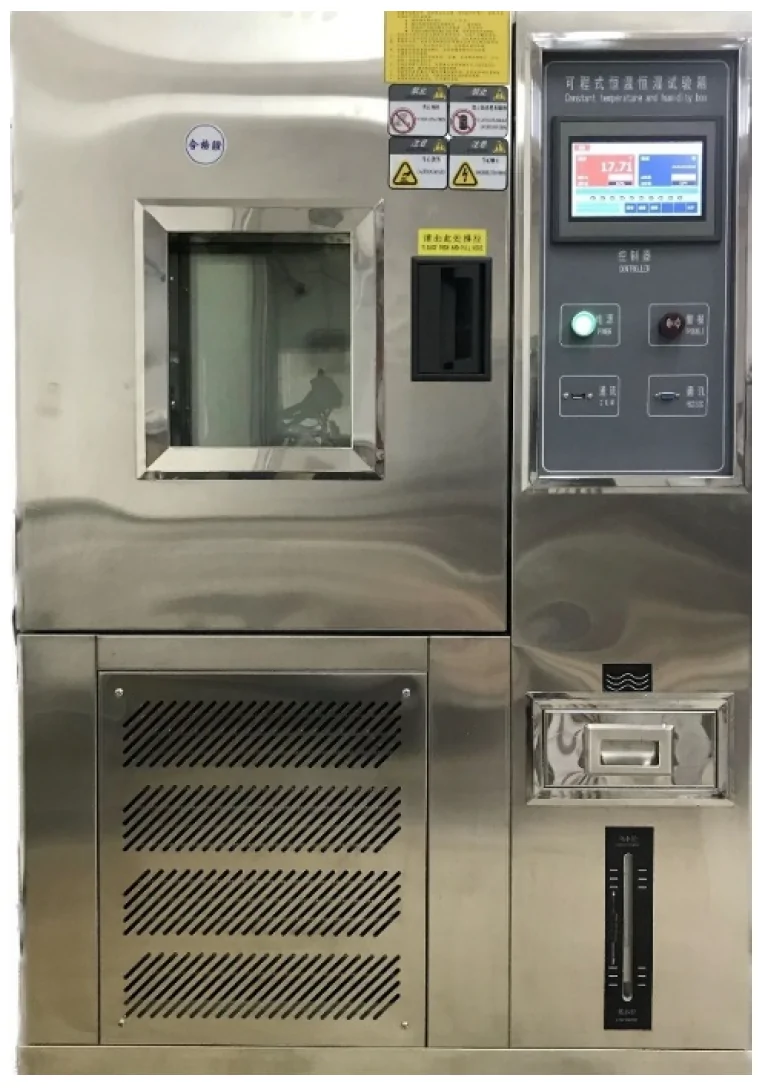
Programmable temperature and humidity chamber.

As shown in Figure 3, freeze–thaw treatment was conducted using an HS-1000 programmable constant temperature and humidity chamber (Heson Instrument Co., Ltd., Shanghai, China), and each freeze–thaw cycle lasted 8 h. The chamber temperature was decreased from 20 °C to −20 °C within 0.5 h and then maintained at −20 °C for 3.5 h. It was subsequently increased from −20 °C to 20 °C within 0.5 h and maintained at 20 °C for 3.5 h. The load was applied before the start of freeze–thaw cycling and kept constant throughout the entire freeze–thaw process. The load–freeze–thaw coupling test scheme is summarized in Table 1. After the preset number of cycles was reached, the sustained load was removed, and the subsequent mechanical-testing procedure was initiated immediately without an additional unloaded holding period. The sustained load was applied during freeze–thaw treatment, whereas its removal before mechanical testing ensured consistent boundary conditions for evaluating the residual mechanical properties of the different groups.

### 2.3. Mechanical Tests

**Figure 4:**
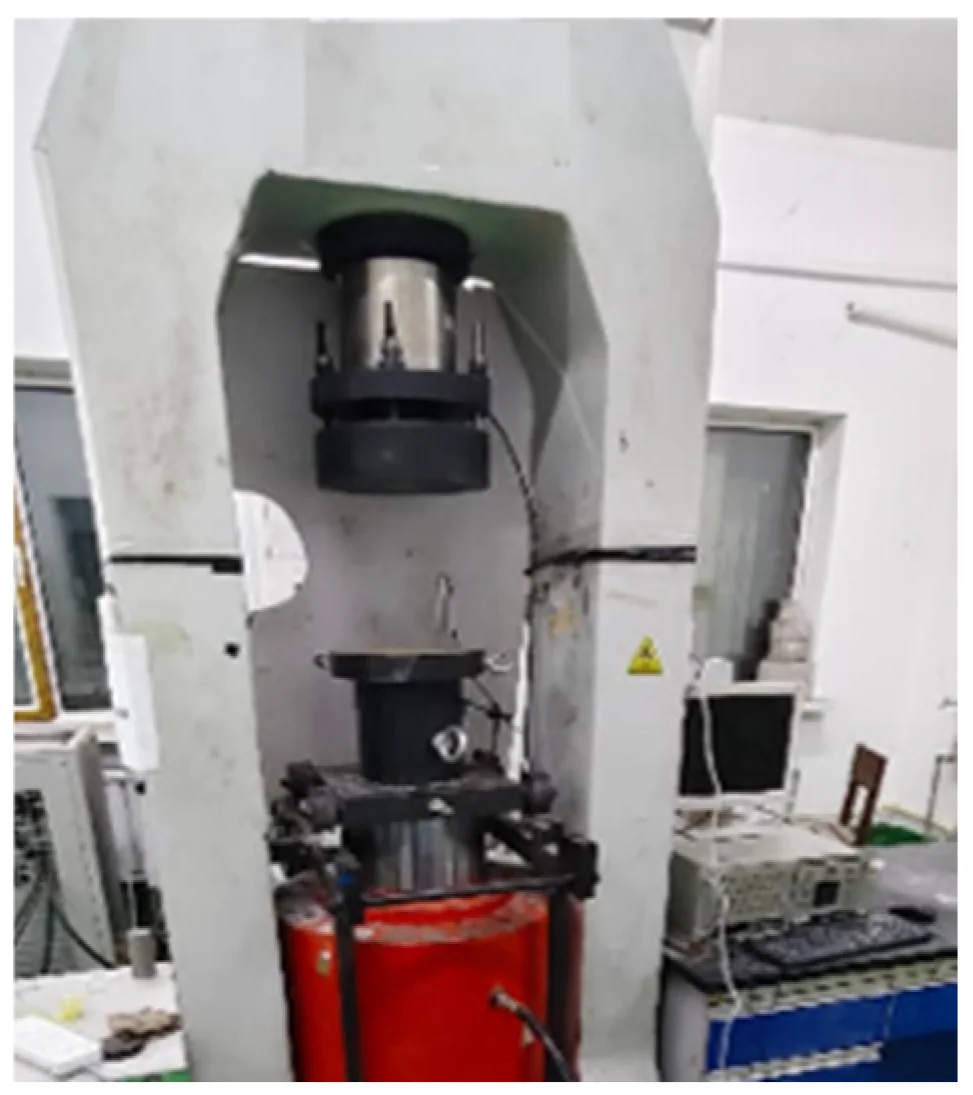
YAW-3000 computer-controlled electro-hydraulic servo testing machine.

For each load–freeze–thaw condition, three specimens were used for the uniaxial compression tests, and three specimens were used for the Brazilian splitting tests, and all specimens were prepared from the same batch of sandstone. The specimens used for the uniaxial compression tests retained the cylindrical dimensions adopted during the load–freeze–thaw treatment. After load–freeze–thaw treatment, the specimens used for the Brazilian splitting tests were processed into disc specimens with a diameter of 50 mm and a thickness of 25 mm. The deviations in disc diameter and thickness were both controlled within ±0.3 mm, and the flatness deviation of the two end faces did not exceed ±0.5 mm. As shown in Figure 4, the uniaxial compression and Brazilian splitting tests were conducted using a YAW-3000 computer-controlled electro-hydraulic servo testing machine (Jilin Jinli Test Technology Co., Ltd., Changchun, China). Both tests were performed under displacement control at a loading rate of 0.002 mm/s. The strength values reported in the following sections are the arithmetic means of the results obtained from three specimens under each condition, whereas the stress–strain curves are representative curves selected from the three tests based on their proximity to the corresponding mean strength and their overall curve characteristics.

### 2.4. Data Processing and Regression Method

The peak stresses obtained from the uniaxial compression and Brazilian splitting tests were taken as the uniaxial compressive strength and tensile strength of sandstone, respectively. To eliminate the influence of the difference in magnitude between the two strength indices and quantitatively compare the strength degradation of sandstone under different load–freeze–thaw conditions, the strength retention rate was defined based on the initial strength of the unfrozen-thawed specimens as follows:

(1) $$R_{i}(N,\sigma_{d})=\frac{\sigma_{i}(N,\sigma_{d})}{\sigma_{i0}},$$

The corresponding strength degradation rate was defined as

(2) $$D_{i}(N,\sigma_{d})=\left[1-\frac{\sigma_{i}(N,\sigma_{d})}{\sigma_{i0}}\right]\times 100\%,$$

where *i* = *c* and *i* = *t* denote uniaxial compressive strength and tensile strength, respectively; *N* is the number of freeze-thaw cycles; $\sigma_{d}$ is the sustained axial stress applied during freeze-thaw cycling; $\sigma_{i}(N,\sigma_{d})$ is the experimental strength under the corresponding condition; and $\sigma_{i0}$ is the initial strength of the unfrozen-thawed specimen. In this study, the initial uniaxial compressive strength $\sigma_{c0}$ was 55.721 MPa, and the initial tensile strength $\sigma_{t0}$ was 3.30 MPa. A smaller strength retention rate $R_{i}$ or a larger strength degradation rate $D_{i}$ indicates a higher degree of strength degradation of sandstone.

To describe the combined effects of the number of freeze–thaw cycles and sustained axial stress on sandstone strength, two-variable exponential regression was performed for the uniaxial compressive strength and tensile strength, with *N* and $\sigma_{d}$ as independent variables. The exponential form was adopted as an empirical representation because the measured strengths exhibited cumulative and nonlinear attenuation with increasing freeze–thaw cycles. This form also satisfies the initial-strength condition at *N* = 0 and allows the strength attenuation rate to vary continuously with sustained stress. The general form of the regression model is

(3) $$\sigma_{i}(N,\sigma_{d})=\sigma_{i0}\exp\left[\left(a_{i}\frac{\sigma_{d}}{\sigma_{c0}}-b_{i}\right)N\right],$$

where $\frac{\sigma_{d}}{\sigma_{c0}}$ is the dimensionless sustained stress level; $a_{i}$ is the sustained-load influence coefficient, which characterizes the regulatory effect of loading on the freeze-thaw strength attenuation rate; and $b_{i}$ is the freeze-thaw attenuation coefficient under no-load conditions. When *N* = 0, the model degenerates to $\sigma_{i}=\sigma_{i0}$, ensuring that the fitted surface passes through the initial strength point of the unfrozen-thawed specimen. When $\sigma_{d}=0$, the model reflects the exponential attenuation of strength with increasing cycle number under single freeze-thaw action. The compressive strength and tensile strength were fitted independently, and the model parameters were determined using nonlinear least squares.

The coefficient of determination $R^{2}$ was used to evaluate the ability of the model to explain the overall variation trend of the experimental data:

(4) $$R^{2}=1-\frac{\sum_{j=1}^{m}{\left(\sigma_{ij}-{\hat{\sigma }}_{ij}\right)}^{2}}{\sum_{j=1}^{m}{\left(\sigma_{ij}-{\bar{\sigma }}_{i}\right)}^{2}},$$

where *m* is the number of test conditions; $\sigma_{ij}$ and ${\hat{\sigma }}_{ij}$ are the experimental and fitted values under the j-th condition, respectively; and ${\bar{\sigma }}_{i}$ is the mean experimental strength of all conditions. A value of $R^{2}$ closer to 1 indicates a better fit of the model to the experimental data.

To further evaluate the local fitting deviation of the model under different conditions, the relative error of each test point was calculated as

(5) $$E_{ij}=\frac{\left|{\hat{\sigma }}_{ij}-\sigma_{ij}\right|}{\sigma_{ij}}\times 100\%.$$

On this basis, the average relative error and maximum relative error of the compressive strength and tensile strength models were calculated separately to comprehensively evaluate the fitting accuracy of the models. The model parameters, fitted surfaces, and error statistics are analyzed in Section 3 together with the experimental results.

## 3. Experimental Results

### 3.1. Uniaxial Compressive Strength Degradation

**Figure 5:**
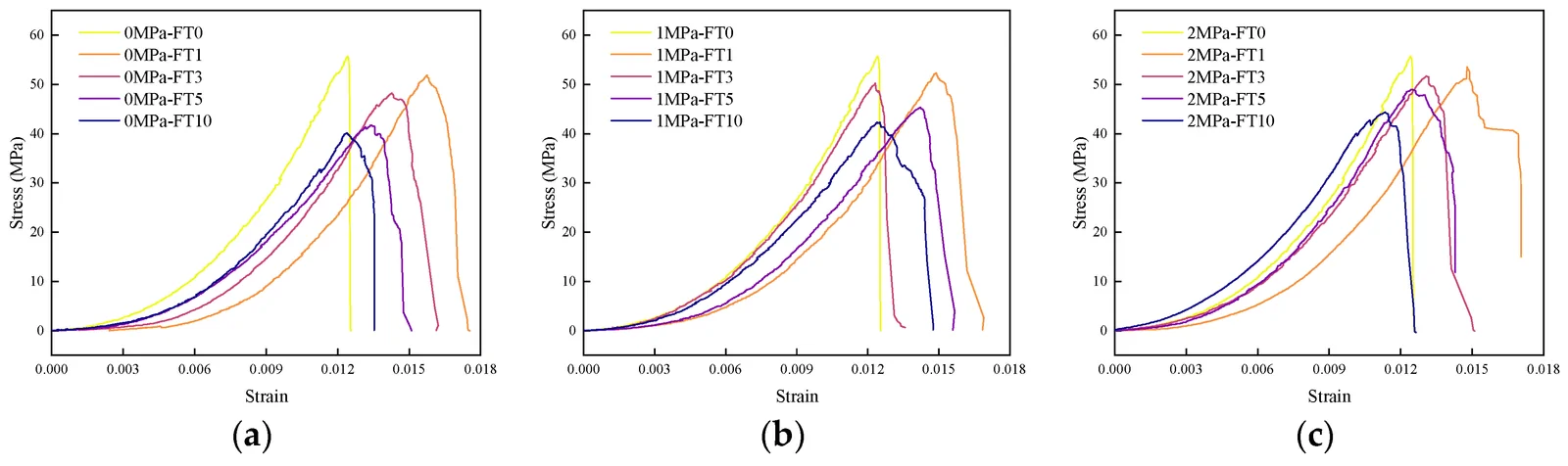
Uniaxial compressive stress–strain curves of sandstone under different loading conditions: (**a**) 0 MPa; (**b**) 1 MPa; (**c**) 2 MPa.

The uniaxial compressive stress–strain curves of sandstone under different freeze–thaw and loading conditions are shown in Figure 5. In the figure labels, FT denotes freeze–thaw, and FTN indicates that the specimen underwent N freeze–thaw cycles. UCS and BTS denote the uniaxial compressive strength and Brazilian tensile strength, respectively. The overall shapes of the curves are similar for all groups. The axial stress increased with strain to the peak value and then dropped rapidly, indicating obvious brittle failure characteristics. With increasing freeze–thaw cycles, the peak stress generally decreased, whereas the peak strain did not exhibit a strictly monotonic variation pattern.

**Figure 6:**
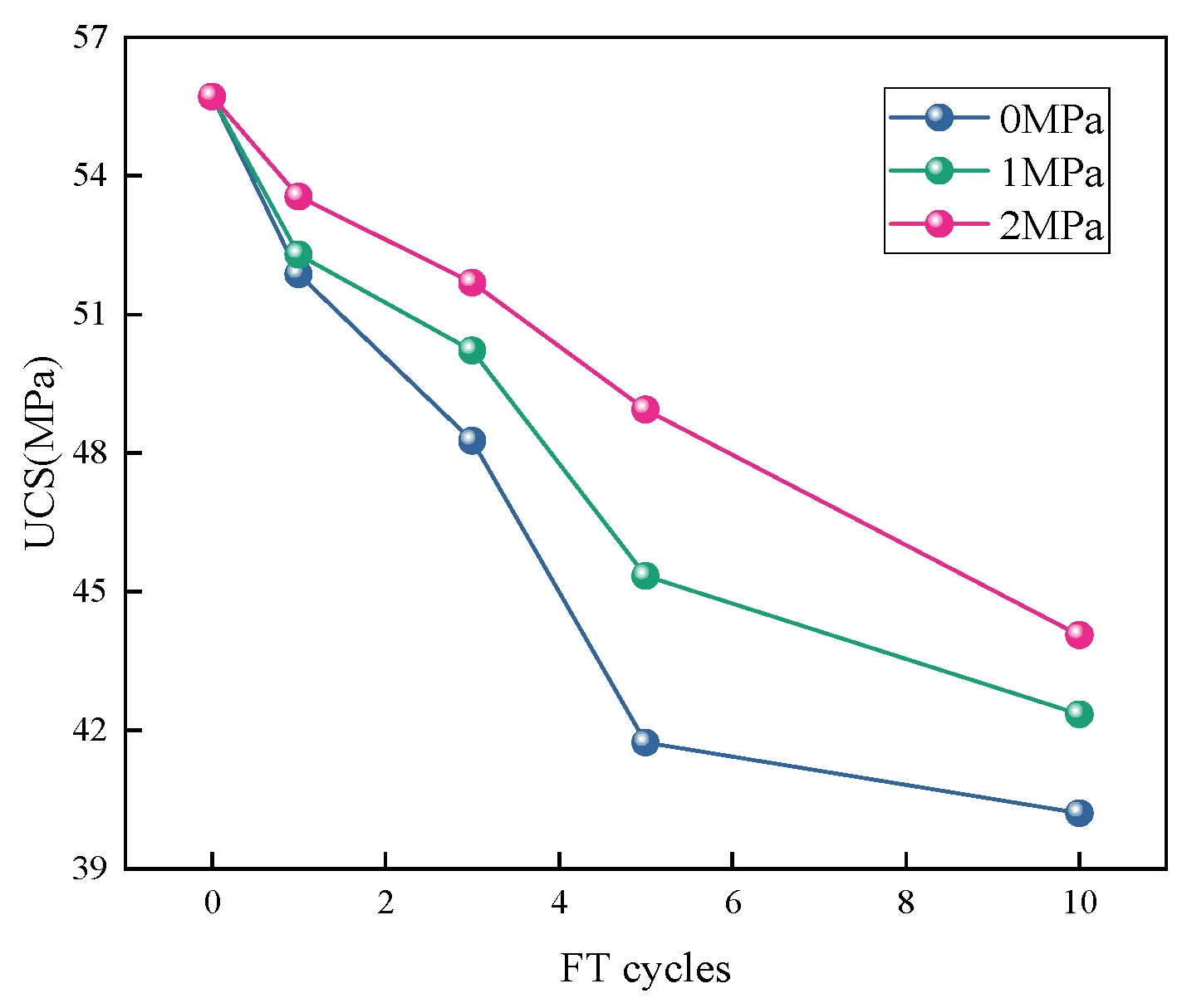
Uniaxial compressive strength of sandstone under different freeze–thaw and loading conditions.

The above uniaxial compressive strengths are summarized in Figure 6. Under the no-load condition, the uniaxial compressive strength of sandstone decreased from 55.721 MPa before freeze–thaw treatment to 51.872, 48.267, 41.730, and 40.202 MPa after 1, 3, 5, and 10 freeze–thaw cycles, respectively, corresponding to strength degradation rates of 6.91%, 13.38%, 25.11%, and 27.85%. The decreases in strength during 0–1, 1–3, 3–5, and 5–10 freeze–thaw cycles were 3.849, 3.605, 6.537, and 1.528 MPa, respectively. Among them, the reduction in compressive strength was most pronounced during 3–5 freeze–thaw cycles, whereas the attenuation became significantly smaller during 5–10 freeze–thaw cycles, indicating that the uniaxial compressive strength of sandstone exhibited a staged nonlinear attenuation with increasing freeze–thaw cycles.

After loading was applied, the uniaxial compressive strength still decreased with increasing freeze–thaw cycles, but the strength at the same number of freeze–thaw cycles increased to some extent. After 10 freeze–thaw cycles, the uniaxial compressive strengths of the 0, 1, and 2 MPa groups were 40.202, 42.341, and 44.058 MPa, respectively, which were 27.85%, 24.01%, and 20.93% lower than that of the unfrozen-thawed specimen. Compared with the no-load group, the compressive strength degradation rate of the 2 MPa group decreased by 6.92%, indicating that loading can mitigate the loss of compressive strength induced by freeze–thaw cycling.

The effect of loading on the compressive strength varied with the number of freeze–thaw cycles. After 1, 3, 5, and 10 freeze–thaw cycles, the uniaxial compressive strength of the 2 MPa group was 3.24%, 7.09%, 17.28%, and 9.59% higher, respectively, than that of the no-load group at the corresponding freeze–thaw cycle. The strength increment reached its maximum after five freeze–thaw cycles and then decreased. The retention effect of loading on compressive strength did not increase monotonically with the number of freeze–thaw cycles but instead exhibited a staged characteristic of first increasing and then decreasing.

### 3.2. Tensile Strength Degradation

**Figure 7:**
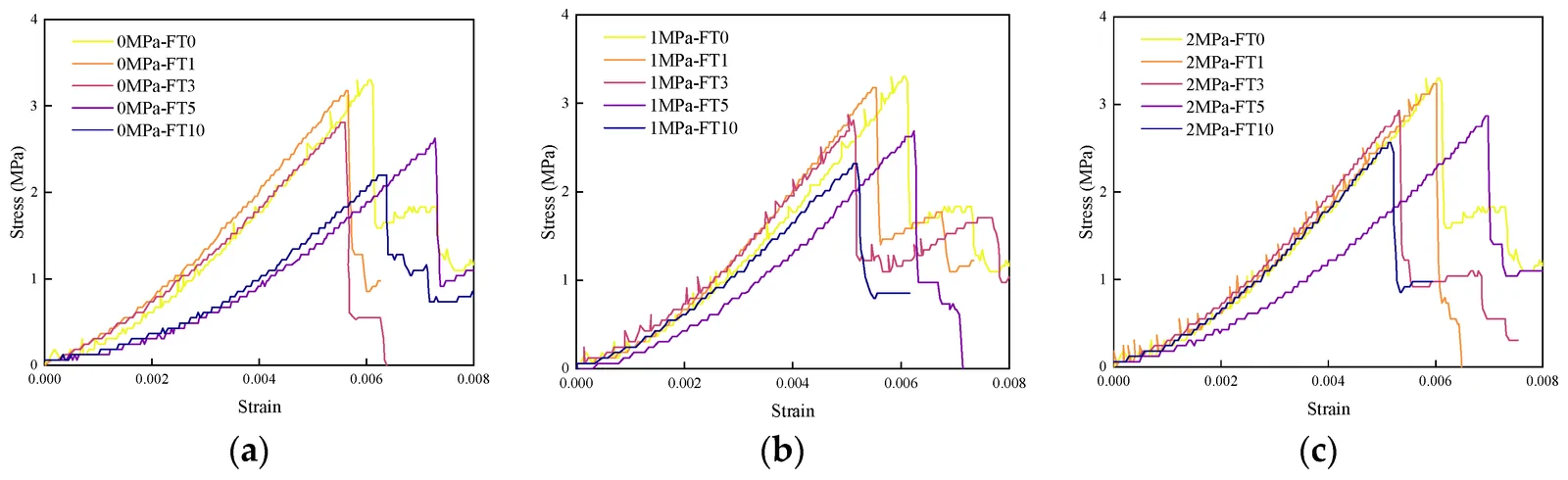
Brazilian splitting stress–strain curves of sandstone under different loading conditions: (**a**) 0 MPa; (**b**) 1 MPa; (**c**) 2 MPa.

The Brazilian splitting stress–strain curves of sandstone under different freeze–thaw and loading conditions are plotted in Figure 7. Overall, the curves of all groups experienced four stages: initial slow increase, nearly linear increase, pre-peak nonlinear deformation, and rapid post-peak decline. After reaching the peak stress, most curves showed a pronounced stress drop, indicating rapid splitting failure of the specimens. The specimens generally exhibited strong brittle characteristics, while the post-peak curves showed different degrees of stress fluctuation and residual bearing capacity.

With increasing freeze–thaw cycles, the peak stress of each loading group generally decreased, and the slope of the pre-peak curves showed a decreasing trend to some extent. This indicates that freeze–thaw damage not only weakened the tensile strength of sandstone but also reduced the overall deformation stiffness. In particular, the curves after 5 and 10 freeze–thaw cycles were generally lower than those of the unfrozen-thawed and early freeze–thaw specimens. However, the peak strain fluctuated under different conditions and did not show a strictly monotonic variation pattern. At the same number of freeze–thaw cycles, the peak stresses of the 1 MPa and 2 MPa groups were generally higher than those of the no-load group, and this difference became more evident at higher numbers of freeze–thaw cycles.

**Figure 8:**
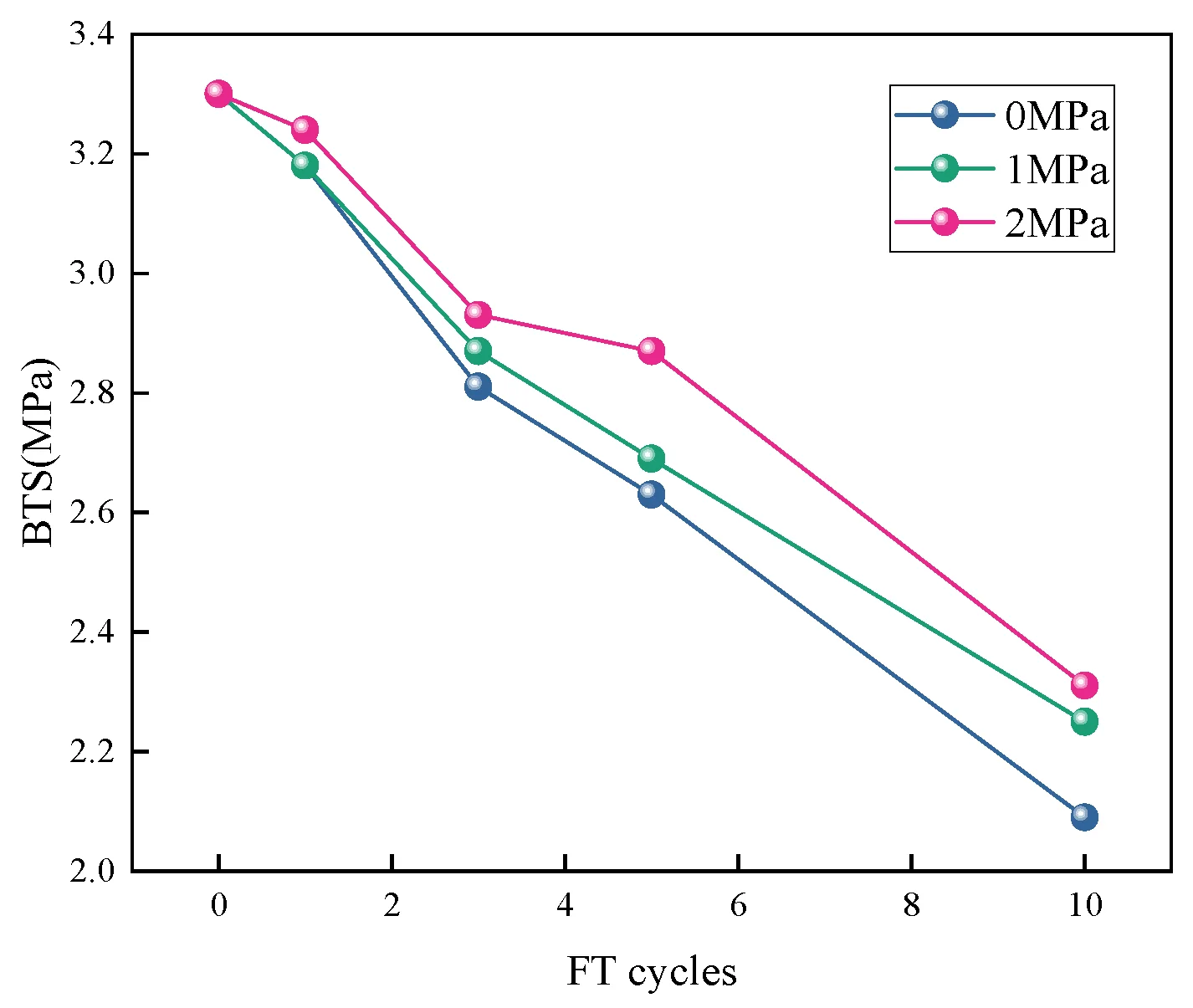
Brazilian tensile strength of sandstone under different freeze–thaw and loading conditions.

The tensile strengths obtained from the Brazilian splitting tests are summarized in Figure 8. Under the no-load condition, the Brazilian tensile strength of sandstone decreased from 3.30 MPa before freeze–thaw treatment to 3.18, 2.81, 2.63, and 2.09 MPa after 1, 3, 5, and 10 freeze–thaw cycles, respectively. The corresponding strength degradation rates were 3.64%, 14.85%, 20.30%, and 36.67%. After one freeze–thaw cycle, the tensile strength decreased only slightly. With increasing freeze–thaw cycles, the strength loss gradually increased. In particular, during 5–10 freeze–thaw cycles, the tensile strength decreased from 2.63 MPa to 2.09 MPa, with a reduction of 20.53%, indicating that the tensile bearing capacity of sandstone continued to deteriorate with freeze–thaw accumulation.

Loading still had an inhibitory effect on the freeze–thaw damage of sandstone tensile strength. After 10 freeze–thaw cycles, the tensile strengths of the 0, 1, and 2 MPa groups were 2.09, 2.25, and 2.31 MPa, respectively, which were 36.67%, 31.82%, and 30.00% lower than that of the unfrozen-thawed specimen. Sustained loading at 1 MPa and 2 MPa increased the tensile strength by 7.66% and 10.53%, respectively, compared with the no-load group.

It can also be observed that loading had only a limited effect on the tensile strength in the early stage of freeze–thaw cycling. After one freeze–thaw cycle, the tensile strengths of the 0 MPa and 1 MPa groups were both 3.18 MPa, while that of the 2 MPa group was 3.24 MPa. With increasing freeze–thaw cycles, the strength difference among different loading groups gradually increased. Compared with the no-load group at the same number of freeze–thaw cycles, the tensile strength increment of the 2 MPa group increased from 1.89% after one freeze–thaw cycle to 4.27%, 9.13%, and 10.53% after three, five, and ten freeze–thaw cycles, respectively.

### 3.3. Effect of Load on Freeze–Thaw Degradation

**Figure 9:**
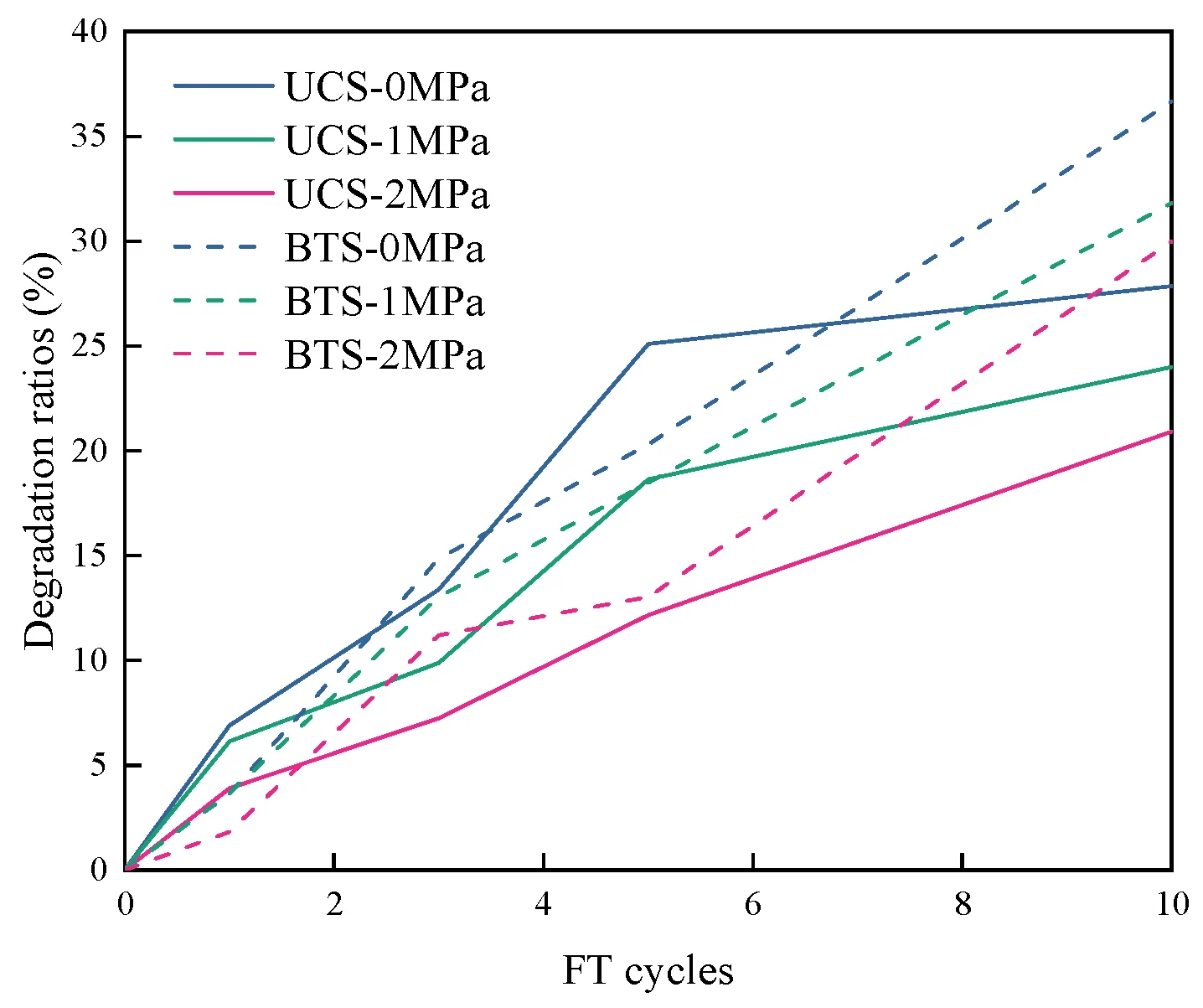
Variations in sandstone strength degradation ratio with freeze–thaw cycles under different loading conditions.

To uniformly compare the freeze–thaw degradation degrees of the compressive and tensile properties of sandstone, the strength degradation rates under different loading conditions were calculated according to Equation (2), as shown in Figure 9. The degradation rates of both the compressive and tensile strengths increased with the number of freeze–thaw cycles, indicating an obvious cumulative effect of freeze–thaw damage. At the same number of freeze–thaw cycles, the strength degradation rate generally decreased with an increasing loading level, suggesting that loading can mitigate the strength loss induced by freeze–thaw cycling.

The responses of the compressive and tensile properties of sandstone to load–freeze–thaw coupling were not completely consistent. Under the no-load condition, after 10 freeze–thaw cycles, the degradation rates of uniaxial compressive strength and tensile strength reached 27.85% and 36.67%, respectively, indicating that the tensile property was more sensitive to freeze–thaw action. After sustained loading was applied, the degradation rates of both strengths decreased, but their staged variations were different. For uniaxial compressive strength, the inhibitory effect of sustained loading was more pronounced in the middle stage of freeze–thaw cycling. Compared with the no-load group at the same number of freeze–thaw cycles, the strength increment of the 2 MPa group increased from 3.24% after one freeze–thaw cycle to 17.28% after five freeze–thaw cycles and then decreased to 9.59% after ten freeze–thaw cycles, showing a characteristic of first increasing and then decreasing. In contrast, the tensile strength showed a more cumulative response to sustained loading. Compared with the no-load group, the strength increment of the 2 MPa group increased gradually from 1.89% after one freeze–thaw cycle to 4.27%, 9.13%, and 10.53% after three, five, and ten freeze–thaw cycles, respectively, indicating that the inhibitory effect of loading on the tensile strength degradation gradually increased with the number of freeze–thaw cycles.

**Figure 10:**
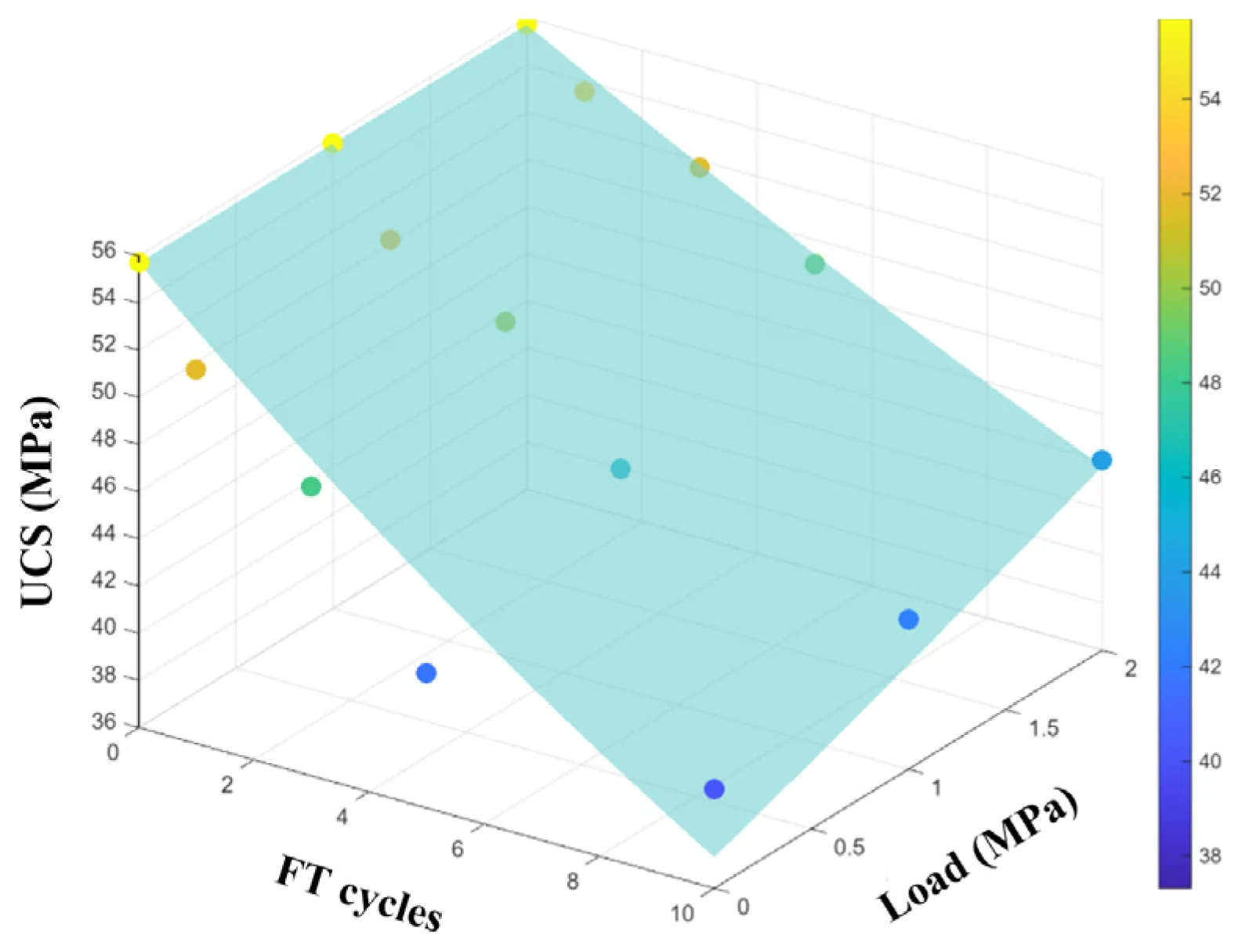
Three-dimensional fitted surface of uniaxial compressive strength of sandstone under load–freeze–thaw coupling.

**Figure 11:**
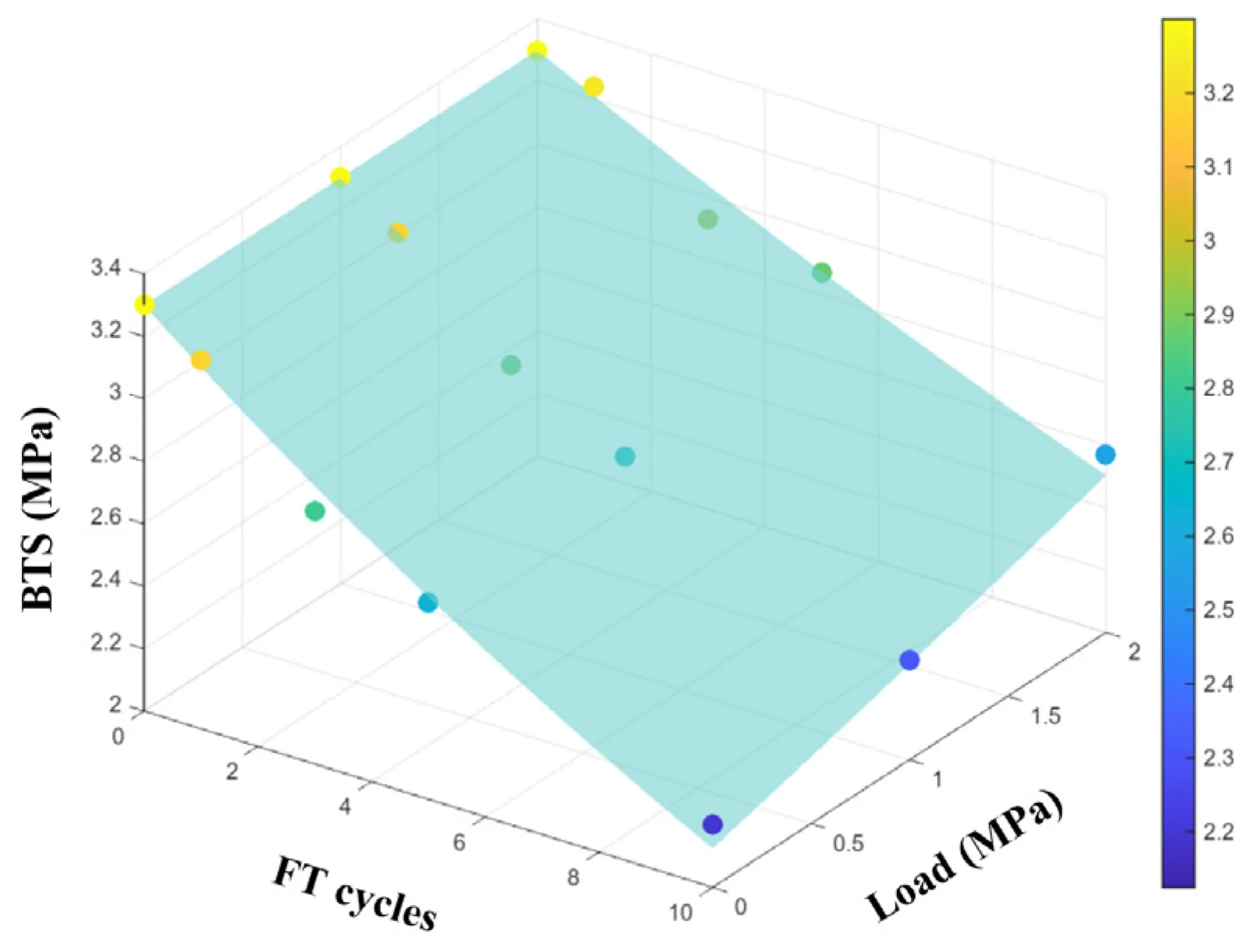
Three-dimensional fitted surface of tensile strength of sandstone under load–freeze–thaw coupling.

To further quantitatively characterize the combined effects of the number of freeze–thaw cycles and sustained axial stress on sandstone strength, two-variable exponential surface fitting was performed for the uniaxial compressive strength and tensile strength according to Equation (3), as shown in Figure 10 and Figure 11. The attenuation model of uniaxial compressive strength of sandstone under load–freeze–thaw coupling can be expressed as

(6) $$\sigma_{c}(N,\sigma_{d})=55.721\exp\left[\left(0.4455\frac{\sigma_{d}}{55.721}-0.0401\right)N\right],$$

where $\sigma_{c}$ is the uniaxial compressive strength, MPa; $\sigma_{d}$ is the sustained axial stress, MPa; and N is the number of freeze-thaw cycles.

The corresponding attenuation model of the tensile strength is

(7) $$\sigma_{t}(N,\sigma_{d})=3.30\exp\left[\left(0.3424\frac{\sigma_{d}}{55.721}-0.0462\right)N\right],$$

where $\sigma_{t}$ is the tensile strength, MPa. Both equations are based on the initial strength of the unfrozen-thawed specimen and can simultaneously describe the cumulative effect of freeze–thaw cycling and the regulatory effect of loading.

According to Equation (4), the coefficients of determination $R^{2}$ of the two strength models were 0.9005 and 0.9896, respectively, indicating that both models can well reflect the overall variation trends of the experimental strengths, with the tensile strength model showing a higher fitting degree. From the physical meaning of the model parameters, the coefficient of the load-related term in the uniaxial compressive strength model was positive, with a value of 0.4455, indicating that sustained loading slowed the attenuation rate of the compressive strength. The constant term of −0.0401 was negative, representing the intrinsic attenuation trend caused by freeze–thaw cycling under no-load conditions. The signs of the two coefficients in the tensile strength model were consistent with those in the compressive strength model, reflecting the same variation pattern. Further comparison of the two sets of parameters shows that the absolute value of the no-load attenuation term in the tensile strength model was larger, corresponding to the experimental result that the tensile property was more sensitive to freeze–thaw action.

To further evaluate the local fitting deviation of the models under different conditions, the fitted values were compared with the measured mean strengths, and the results calculated using Equation (5) are shown in Table 2. Among the 13 test conditions, the average relative error of the uniaxial compressive strength model was 3.0%, and the maximum relative error was 9.2%. Except for one condition, the errors of the other 12 conditions were all within 5%. The average relative error of the tensile strength model was 1.2%, and the maximum relative error was 2.8%, with all conditions controlled within 3%, indicating satisfactory overall fitting accuracy. The two models are intended to describe the strength variation of the sandstone investigated in this study within sustained stresses of 0–2 MPa and 0–10 freeze–thaw cycles. Application to other sandstone types or conditions beyond the investigated ranges requires recalibration and verification using corresponding experimental data.

## 4. Discrete Element Modeling

### 4.1. Numerical Model Construction and Parameter Calibration

A two-dimensional discrete element model of sandstone was established using the Particle Flow Code in Two Dimensions, version 5.0 (PFC2D 5.0). The rectangular specimen was 50 mm × 100 mm, consistent with the specimen used in the uniaxial compression test. The Brazilian splitting model was obtained by cutting a circular region with a diameter of 50 mm from the rectangular parent model, so that the two types of models had the same particle composition and initial contact structure.

**Figure 12:**
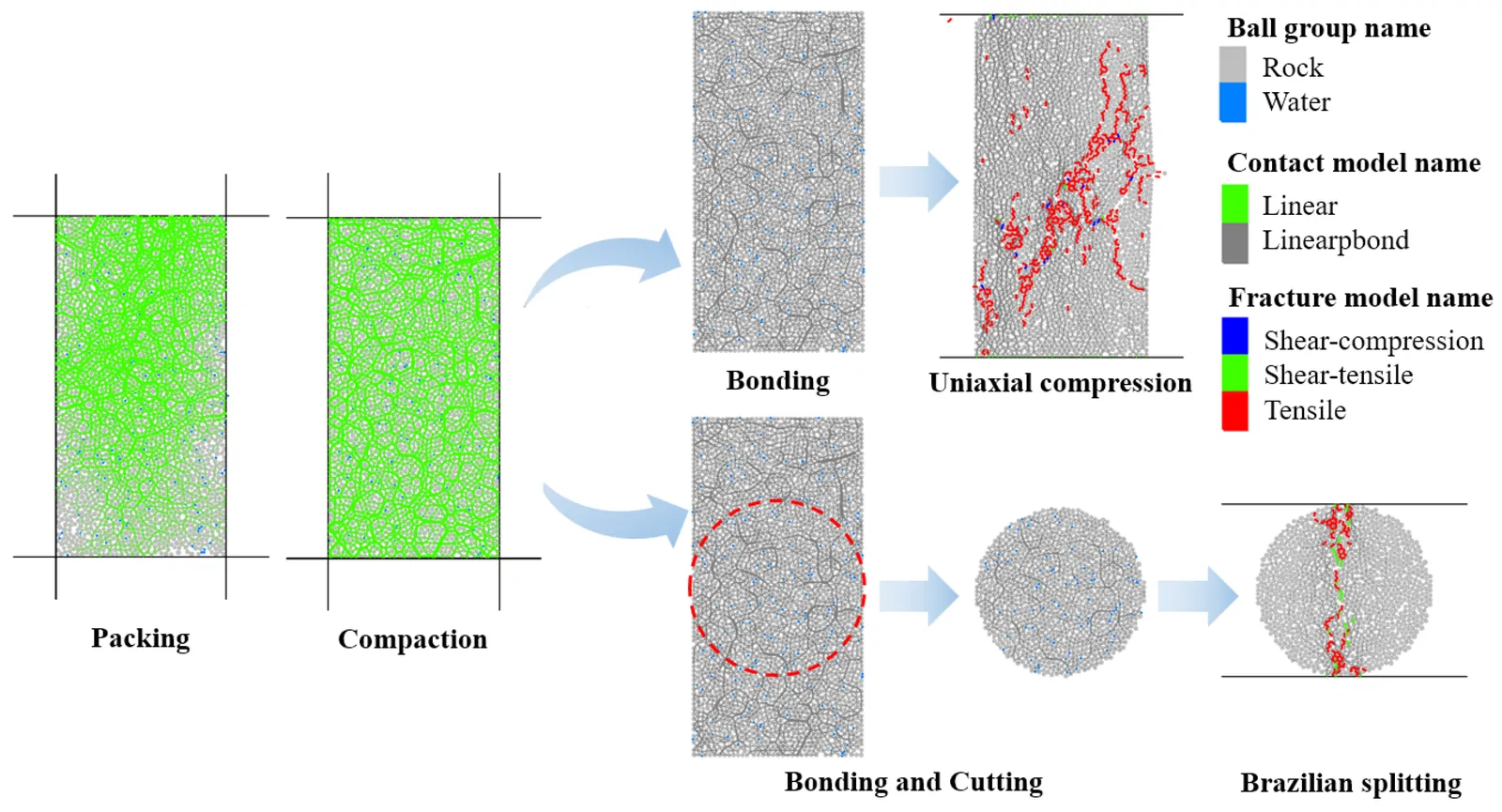
Construction procedure of the DEM models for uniaxial compression and Brazilian splitting tests.

The model construction procedure is shown in Figure 12. Rock particles and equivalent water particles were first randomly generated in the rectangular region. The radius of rock particles ranged from 0.58 to 0.78 mm, and the radius of equivalent water particles was 0.38 mm. The particle-size range was selected to balance the spatial resolution required for crack evolution with the computational efficiency of the coupled thermal-mechanical simulation. Since the particle-size distribution can influence the macroscopic response and fracture development of PFC models [18], the same particle-size distribution was maintained for all loading and freeze–thaw conditions. The proportion of equivalent water particles was set as an equivalent numerical parameter based on the measured saturated mass water content of the sandstone specimens. The particles were rearranged through biaxial compaction to form a stable contact network. After the model reached equilibrium, parallel bonds were installed at particle contacts to form a sandstone model with cementation strength. The bonded rectangular specimen was directly used for uniaxial compression simulation, while the Brazilian splitting specimen was obtained by circular cutting and loaded along the disc diameter for Brazilian splitting simulation. Water particles were deleted before loading in all models.

**Figure 13:**
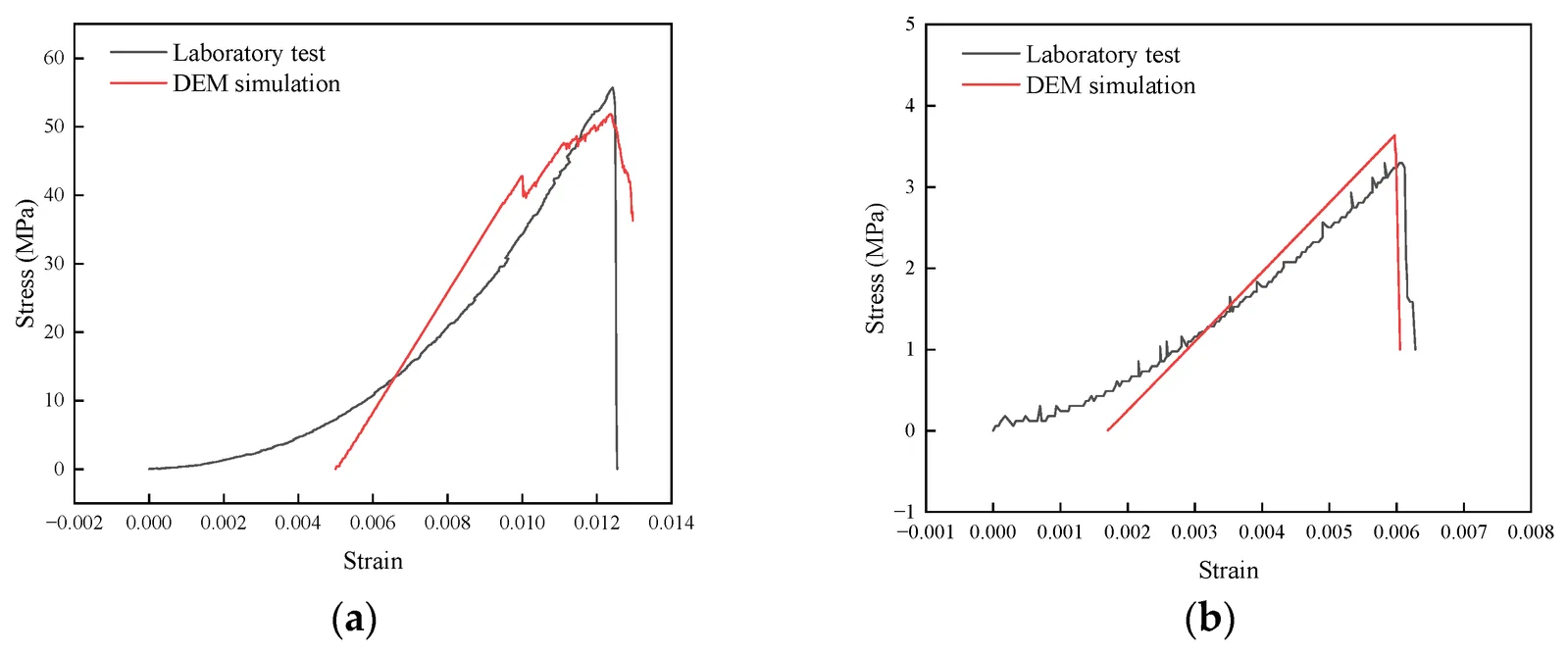
Comparison of experimental and DEM-simulated stress–strain curves of unfrozen-thawed sandstone without sustained loading (0 MPa-FT0): (**a**) uniaxial compression; (**b**) Brazilian splitting.

The microscopic parameters of the model were jointly calibrated using the uniaxial compression and Brazilian splitting responses of the unfrozen-thawed sandstone without sustained loading. An iterative calibration procedure was adopted in which the particle elastic modulus, stiffness ratio, parallel-bond tensile strength, and cohesion were adjusted as a parameter set. Considering that the initial portions of the laboratory curves were affected by the closure of primary pores and the contact between the specimen ends and loading platens, the calibration mainly focused on the principal pre-peak load-bearing stage and peak strengths. The same parameter set was retained when it could reasonably reproduce the principal pre-peak responses and peak strengths of both the uniaxial compression and Brazilian splitting tests. The comparison shown in Figure 13 was then used to verify the calibrated mechanical response of the rock skeleton before the load–freeze–thaw coupled simulations.

The stress–strain curves from the uniaxial compression and Brazilian splitting tests of the unfrozen-thawed specimens without sustained loading (0 MPa-FT0) and the corresponding simulations are plotted in Figure 13. The peak strengths and overall curve trends of the two types of simulations were generally consistent with the laboratory test results, satisfying the basic requirements for the rock skeleton in the subsequent load–freeze–thaw coupled simulations. The water content of the actual sandstone specimens was further tested. Based on the mass difference before and after saturation, the saturated mass water content was calculated using Equation (8) and was approximately 6%.

(8) $$W=\frac{m_{s}-m_{d}}{m_{d}}\times 100\%,$$

where is the saturated mass water content of the specimen, $m_{d}$ is the oven-dried mass of the specimen, and $m_{s}$ is the mass of the specimen after complete saturation.

The numerical simulation was set to correspond to the fully saturated state in the tests. Pore water was represented by equivalent water particles, and the proportion of equivalent water particles was set according to the saturated mass water content of 6%, so as to represent the subsequent frost-heave effect of pore water. The main calibrated microscopic parameters of the model are listed in Table 3. To ensure the stability of the rock–water interface during frost heave, the bond strength between rock particles and water particles was set to 10 times the basic strength listed in the table.

### 4.2. Thermal Field and Frost-Heave Simulation

To simulate the temperature transfer inside sandstone and the frost-heave effect of pore water during freeze–thaw cycling, the thermal calculation module was activated based on the particle mechanical model. The representation of pore water by expandable equivalent particles and the simulation of freeze–thaw damage through bond breakage follow established DEM approaches for rock freeze–thaw modeling [14,15,16,17]. Heat transfer between particles, particle temperature updating, and frost-heave deformation of water particles were carried out simultaneously with the mechanical calculation. When the local action induced by water-particle expansion exceeded the bond strength between rock particles, the parallel bond broke, thereby simulating the irreversible freeze–thaw damage inside real rock. During the simulation, each irreversible parallel-bond break event was monitored using the FISH function and counted once. Bond breaks occurring in the tensile and shear failure modes were classified as tensile cracks and shear–tensile cracks, respectively. The cumulative crack counts were updated at the end of each freeze–thaw cycle.

#### 4.2.1. Heat-Transfer Model and Thermal Boundary Conditions

**Figure 14:**
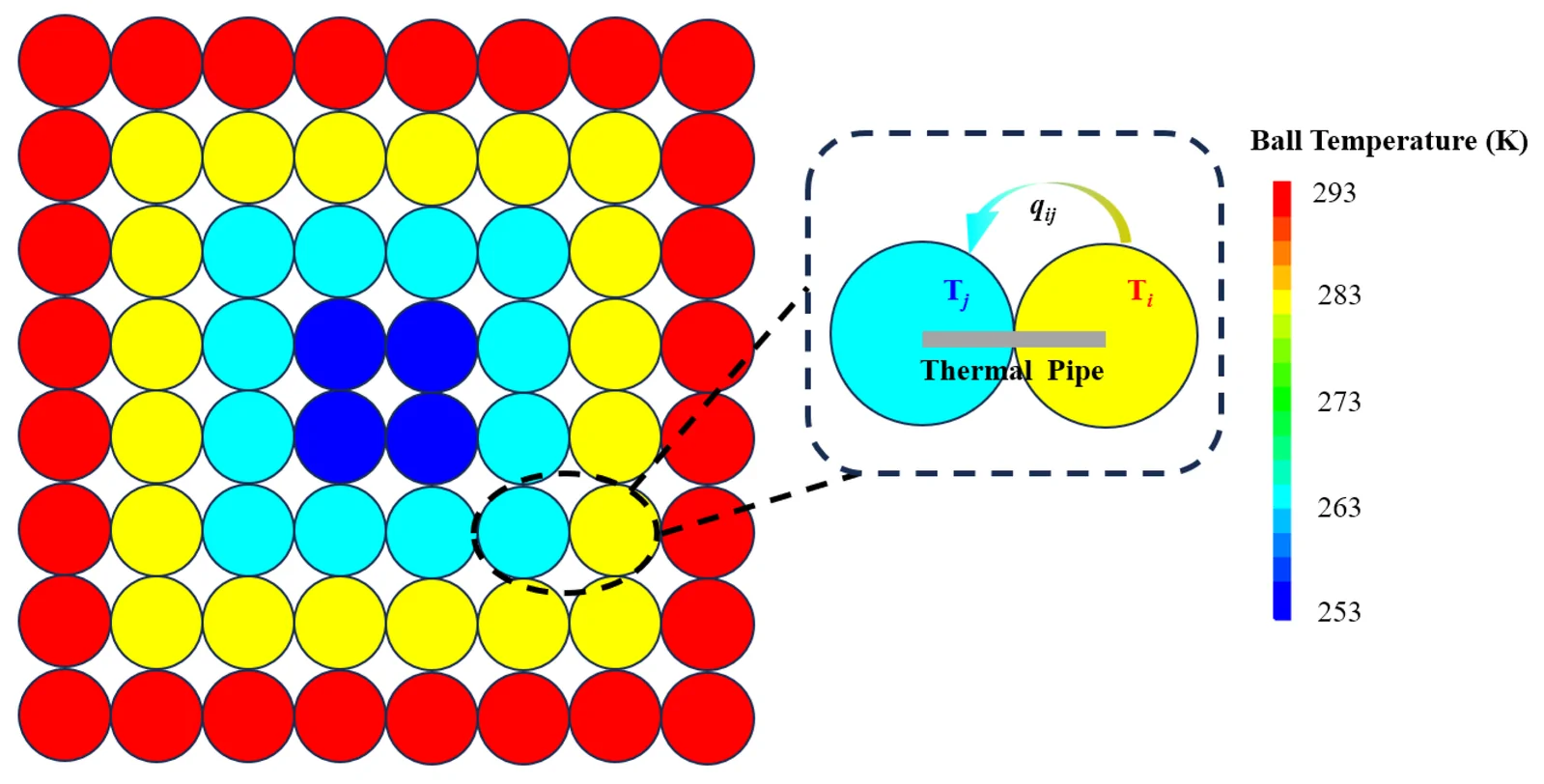
Schematic of heat transfer between particles in the DEM model.

In the heat-transfer calculation of the PFC 5.0 thermal module, each particle is treated as an independent heat-storage unit, and adjacent particles form thermal pipes at their contact positions, as shown in Figure 14. When a temperature difference exists between two particles, heat is transferred from the high-temperature particle to the low-temperature particle through the thermal pipe, and the particle temperature is continuously updated according to the net thermal power. The heat-transfer power between particle *i* and particle *j* can be expressed as

(9) $$Q_{ij}=-\frac{T_{j}-T_{i}}{\eta_{ij}L_{ij}},$$

where $Q_{ij}$ is the thermal power transferred from particle *i* to particle *j*; $T_{i}$ and $T_{j}$ are the temperatures of the two particles, respectively; $\eta_{ij}$ is the thermal resistance of the contact thermal pipe; and $L_{ij}$ is the distance between the centers of the two particles. When $T_{i}>T_{j}$, heat is transferred from particle *i* to particle *j* and gradually diffuses into the model through the particle contact network.

**Figure 15:**
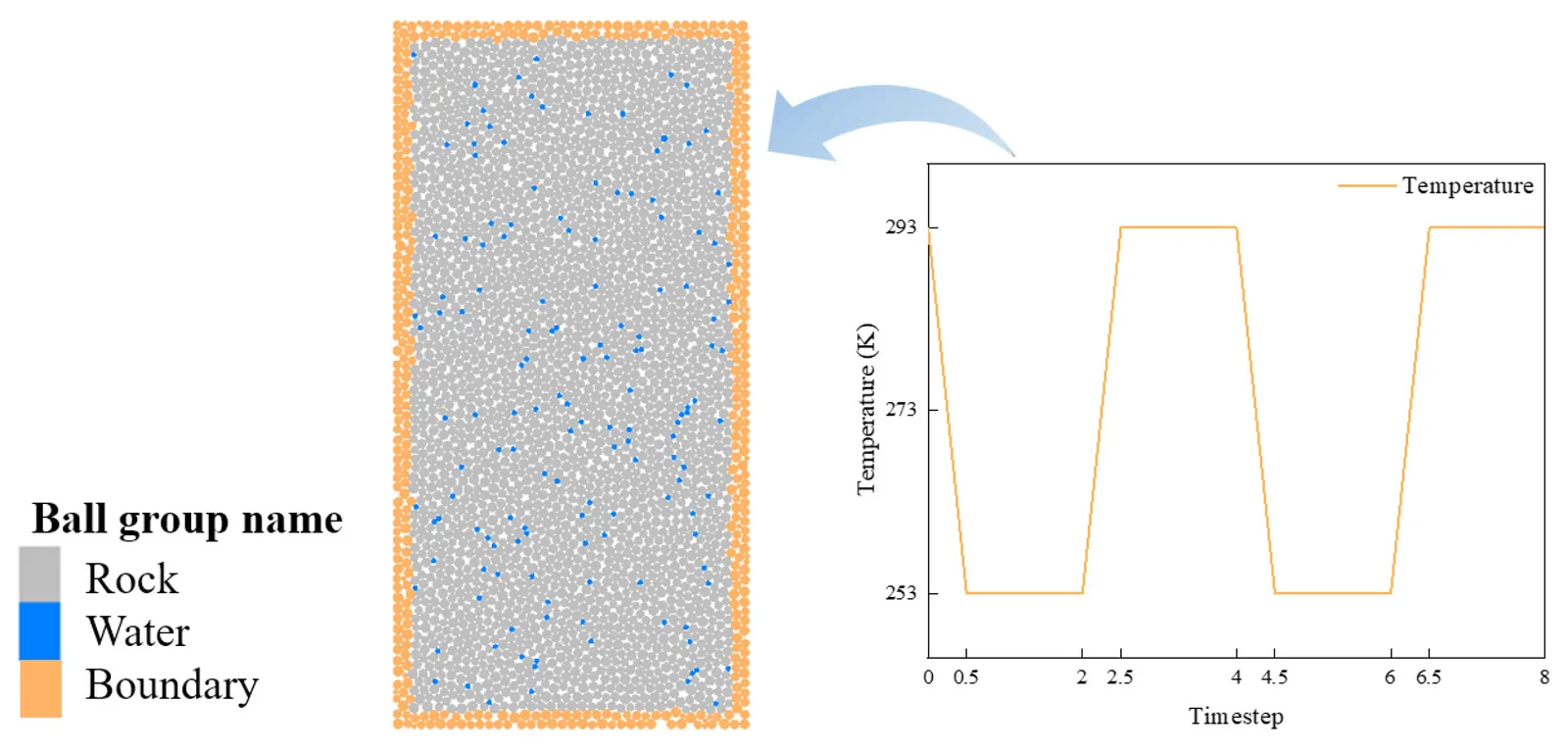
Numerical model and thermal boundary conditions used in the freeze–thaw simulation.

During the freeze–thaw simulation, the initial temperature of all particles was set uniformly to 293 K. Figure 15 shows the particle composition and thermal boundary setting of the numerical model. The orange particles represent the thermal boundary particles, the gray particles represent the sandstone matrix particles, and the blue particles represent the equivalent water particles. These colors distinguish the particle types rather than their temperatures. Particles within 2 mm of the four model boundaries were defined as the thermal boundary layer, and their temperatures were prescribed according to the temperature variation curve shown in Figure 15. A single freeze–thaw cycle consisted of four stages: cooling, low-temperature holding, heating, and high-temperature holding. The overall temperature variation process was generally consistent with the experimental freeze–thaw settings.

The thermal boundary first acted on the peripheral particles of the model and then transferred inward step by step through the thermal pipes between particles, forming a time-dependent nonuniform temperature field inside the specimen. After one cycle, the whole model had basically recovered to 293 K. The next freeze–thaw cycle was then continued while retaining the existing bond-breakage damage, until the corresponding preset number of cycles was reached.

#### 4.2.2. Frost-Heave Expansion and Bond Breakage

The frost-heave effect was simulated by the radial deformation of water particles caused by temperature change. The thermal expansion coefficient of rock particles was set to 0, and the relative radius increment of water particles was calculated as follows:

(10) $$\frac{\Delta r}{r}=\alpha_{w}\Delta T,$$

where r is the current radius of the water particle, Δr is the radius change under the temperature increment, $\alpha_{w}$ is the equivalent expansion coefficient of the water particle, and ΔT is the temperature increment in the current calculation step.

**Figure 16:**
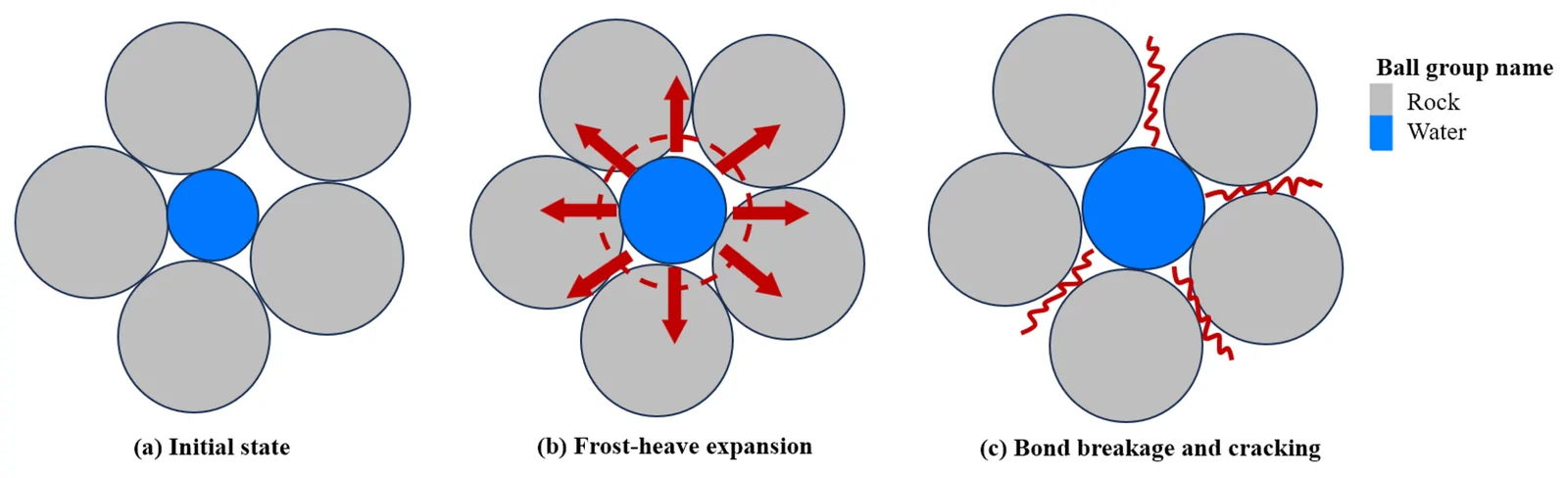
Schematic illustration of frost-heave-induced crack evolution.

The frost-heave and crack formation process of water particles is shown in Figure 16. In the initial state, water particles were distributed in local pores formed by rock particles. As the temperature entered the freezing range, the radius of water particles gradually increased and squeezed the surrounding rock particles, increasing the overlap and contact force at water–rock contacts. The frost-heave force was transmitted to the surrounding area through the particle contact network and produced stress concentration in local parallel bonds. When the maximum stress borne by a parallel bond exceeded its corresponding strength, the bond fractured, and a corresponding discrete crack was generated at the broken contact. The cracks generated during each freeze–thaw cycle were continuously retained and accumulated in the model, thereby representing the evolution of freeze–thaw damage in sandstone from local bond breakage to macroscopic crack propagation.

### 4.3. Load–Freeze–Thaw Coupling Path and Numerical Conditions

For the 1 MPa and 2 MPa load–freeze–thaw coupled models, rigid loading walls were set at the upper and lower boundaries of the model before freeze–thaw simulation. The loading stress was calculated from the average contact force of the upper and lower loading walls:

(11) $$\sigma_{d}=\frac{\left|F_{u}\right|+\left|F_{l}\right|}{2W},$$

where $\sigma_{d}$ is the sustained axial stress, MPa; $F_{u}$ and $F_{l}$ are the axial contact forces of the upper and lower loading walls, respectively, kN; W is the product of the model width and unit thickness, mm^2^.

The 0 MPa group only experienced freeze–thaw cycles during the freeze–thaw process. The 1 MPa and 2 MPa groups were loaded to the target stress before freeze–thaw cycling and were maintained constant throughout the whole freeze–thaw process. After each cycle, the model temperature recovered to 293 K, while the formed particle displacement, contact changes, and bond damage were retained and used as the initial state for the next cycle. By repeating the above process, uniaxial compression models after 0–10 freeze–thaw cycles under different loading conditions were obtained. After the preset number of freeze–thaw cycles was reached, the sustained load was removed, and the model was re-equilibrated. The rectangular model was directly used for uniaxial compression simulation, while the Brazilian splitting model was obtained by cutting a circular region with a diameter of 50 mm from the center of the freeze–thaw-treated rectangular model.

## 5. Numerical Results and Discussion

### 5.1. Thermal Field Evolution

**Figure 17:**
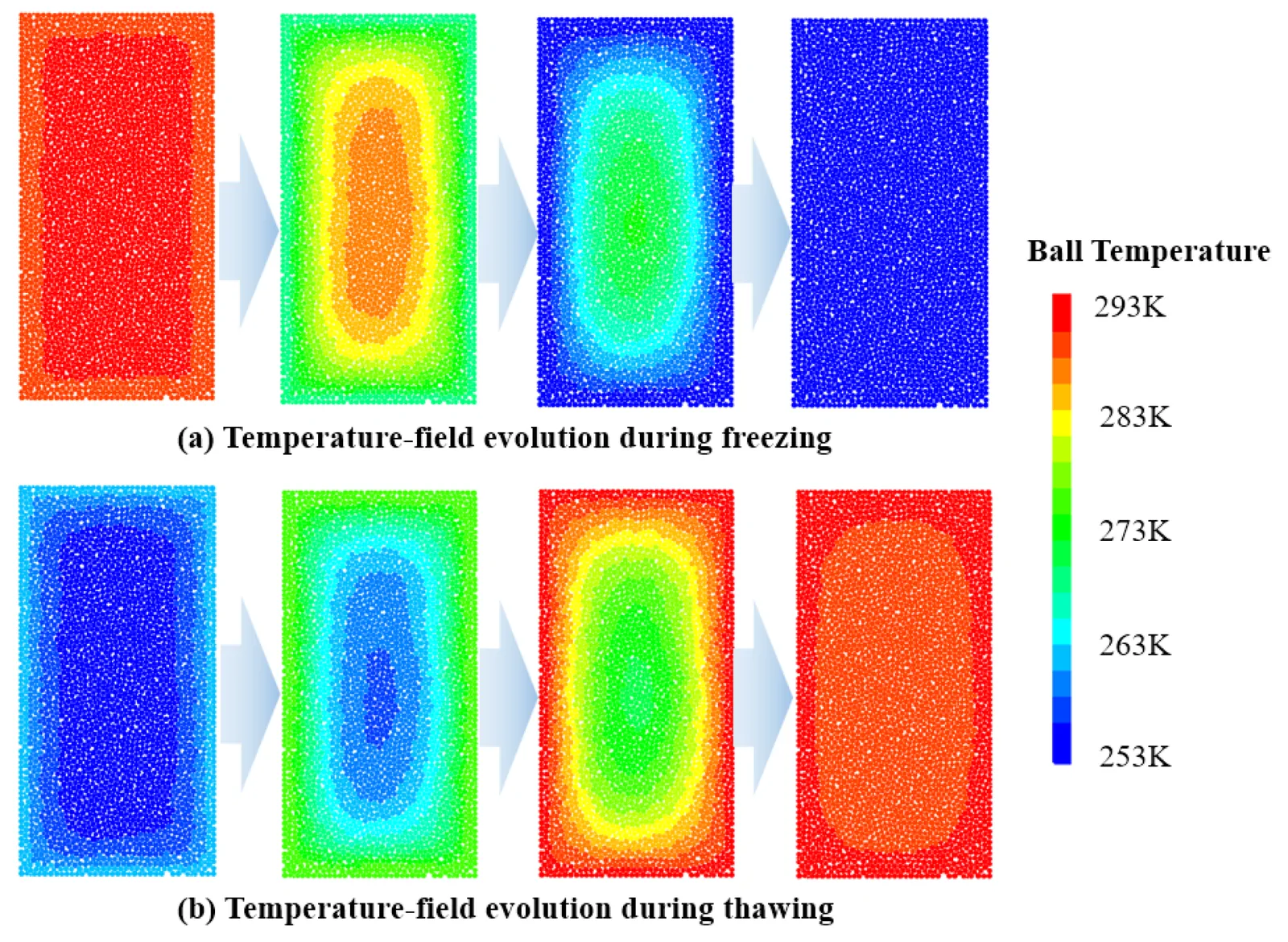
Temperature-field evolution of the sandstone model during a freeze–thaw cycle.

The thermal-field evolution of the sandstone model during a single freeze–thaw cycle is shown in Figure 17. Since the thermal boundary was applied to the four boundaries of the model, the particle temperature first changed at the outer edge and then gradually transferred inward through the thermal pipes formed by particle contacts. Both the freezing and thawing stages showed a temperature propagation pattern from the boundary toward the center, forming a spatial temperature gradient inside the specimen.

After the freezing stage began, the temperature of the peripheral particles decreased first, and the low-temperature region expanded inward along the four boundaries, while the central region still maintained a relatively high temperature. As cooling continued, the low-temperature region gradually enlarged, the temperature difference between the boundary and the center continuously decreased, and finally, the whole model approached the prescribed low-temperature state. During the thawing stage, the direction of heat transfer was the same as that in the freezing stage, but the temperature variation trend was opposite, forming a temperature distribution with higher temperature outside and lower temperature inside. With continuous heat input, the internal low-temperature region gradually shrank until the whole specimen basically recovered to the initial temperature.

During both freezing and thawing, the temperature change at the boundary was not synchronized with that inside the model, because heat needed to be transferred step by step through the particle contact network. The nonuniform temperature field developing from the outside to the inside caused water particles at different positions to enter the frost-heave or thawing range successively, leading to the progressive development of frost-heave action in both space and time. This is also an important feature that distinguishes this method from some DEM simulations in which particle volume expansion is uniformly controlled using a fixed function.

### 5.2. Crack Evolution During Load–Freeze–Thaw Coupling

**Figure 18:**
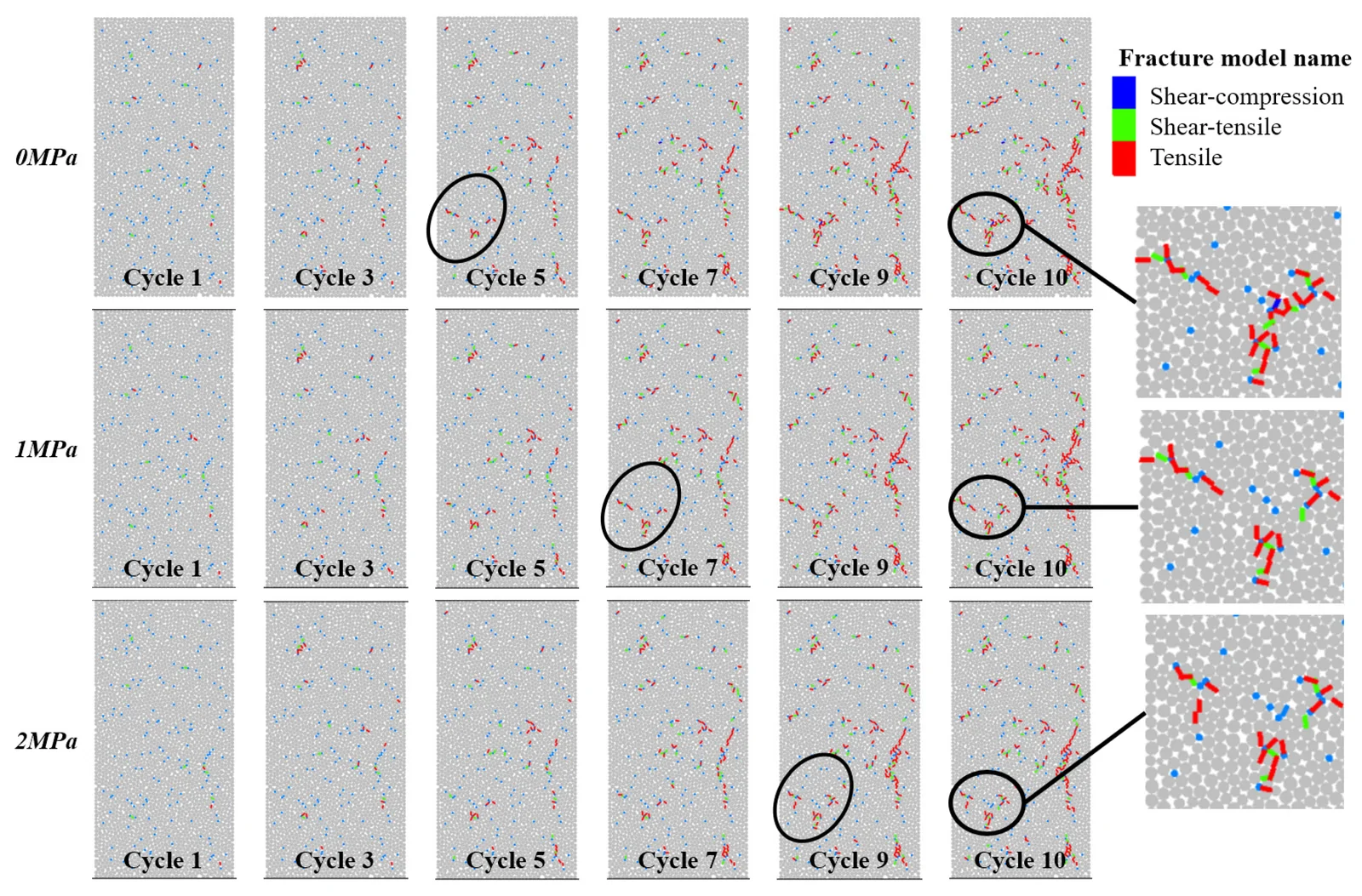
Crack distributions in the sandstone DEM models under different loading conditions and freeze–thaw cycles.

Figure 18 summarizes the crack distributions in the sandstone models under different load–freeze–thaw coupling conditions. Since the same initial particle structure and equivalent water-particle distribution were used for all conditions, the main crack-concentrated regions in the three groups were generally similar, but the crack number and development degree differed to some extent. After one to three freeze–thaw cycles, bond breakage was mainly scattered near the equivalent water particles, and the cracks were short and independent. The differences among different loading groups were not yet obvious. With increasing freeze–thaw cycles, cracks gradually propagated from local initiation sites toward adjacent weak contact regions, and crack connection and clustering appeared. After seven freeze–thaw cycles, relatively obvious crack clusters formed in some regions. By 9–10 freeze–thaw cycles, the crack number increased rapidly, and local cracks further connected and extended, indicating that repeated frost-heave action caused continuous damage accumulation inside sandstone.

At the same number of freeze–thaw cycles, the crack distribution range and local concentration degree in the no-load group were generally higher than those in the loaded groups, and this difference gradually became more evident with increasing freeze–thaw cycles. In the early stage of freeze–thaw cycling, the sustained loads of 1 MPa and 2 MPa did not significantly change the crack distribution. In the middle and later stages, the crack number and clustering degree in the 2 MPa group were lower than those in the 0 MPa and 1 MPa groups. Therefore, loading did not change the main initiation locations of freeze–thaw cracks but limited their continuous propagation and local coalescence.

**Figure 19:**
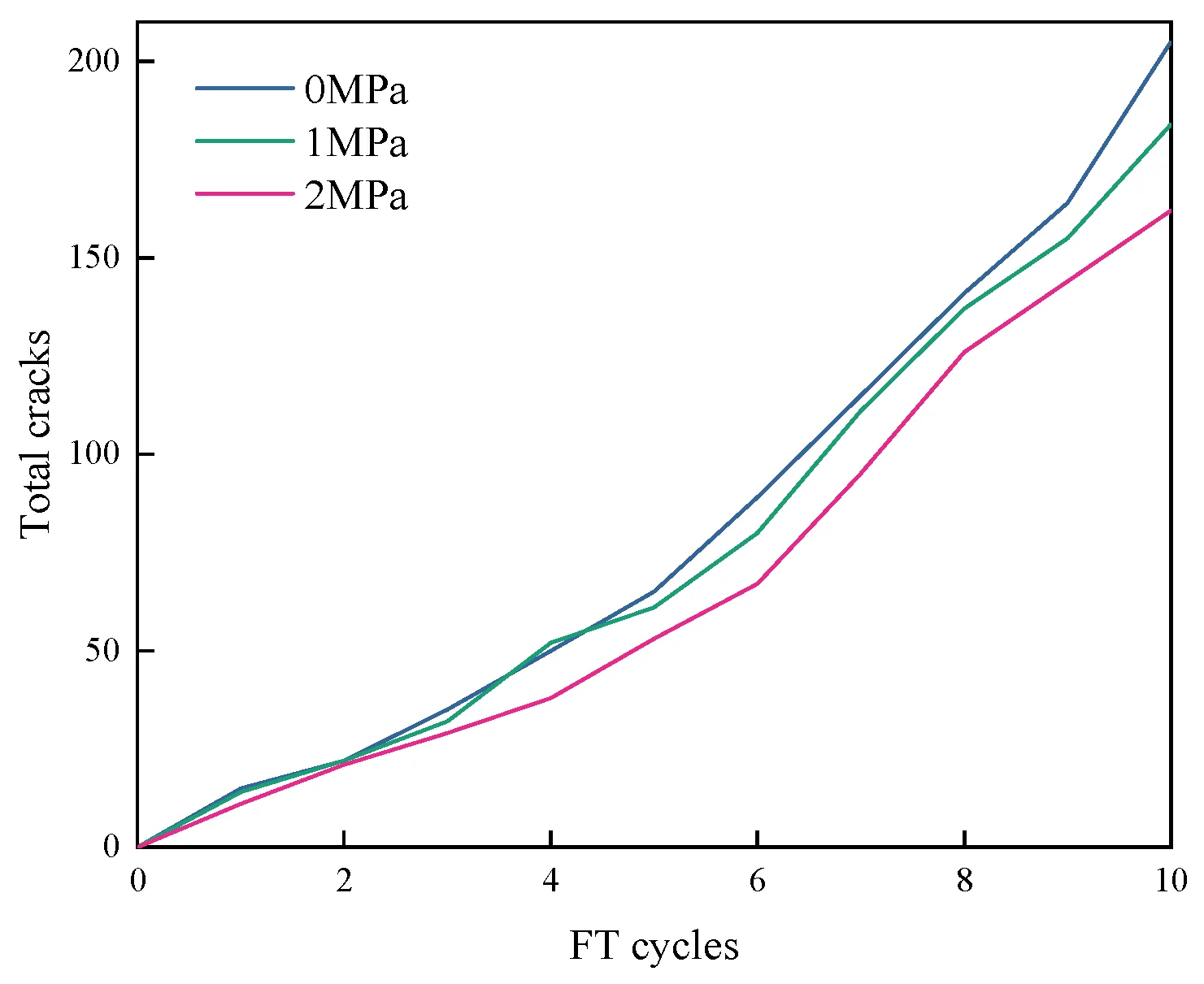
Evolution of the cumulative total crack count in the sandstone DEM models under different loading conditions.

The evolution curves of the cumulative total crack count under different loading conditions are summarized in Figure 19. The total crack count of all three groups continuously increased with the number of freeze–thaw cycles, indicating the obvious cumulative nature of freeze–thaw damage. During the first three cycles, the curves of different groups were relatively close and locally intersected, indicating that loading had a weak influence on initial crack initiation. After four to six freeze–thaw cycles, differences among different loading groups began to develop. After six freeze–thaw cycles, the crack number increased more rapidly and gradually showed a stable relationship, with the 0 MPa group being the highest, followed by the 1 MPa group, and the 2 MPa group being the lowest.

After 10 freeze–thaw cycles, the cumulative total crack counts in the 0, 1, and 2 MPa groups were 205, 183, and 161, respectively. Compared with the no-load group, the 1 MPa and 2 MPa groups decreased by approximately 10.7% and 21.5%, respectively. This indicates that sustained axial compressive stress inhibited crack accumulation after multiple freeze–thaw cycles, and the inhibitory effect was further enhanced when the loading level increased from 1 MPa to 2 MPa.

**Figure 20:**
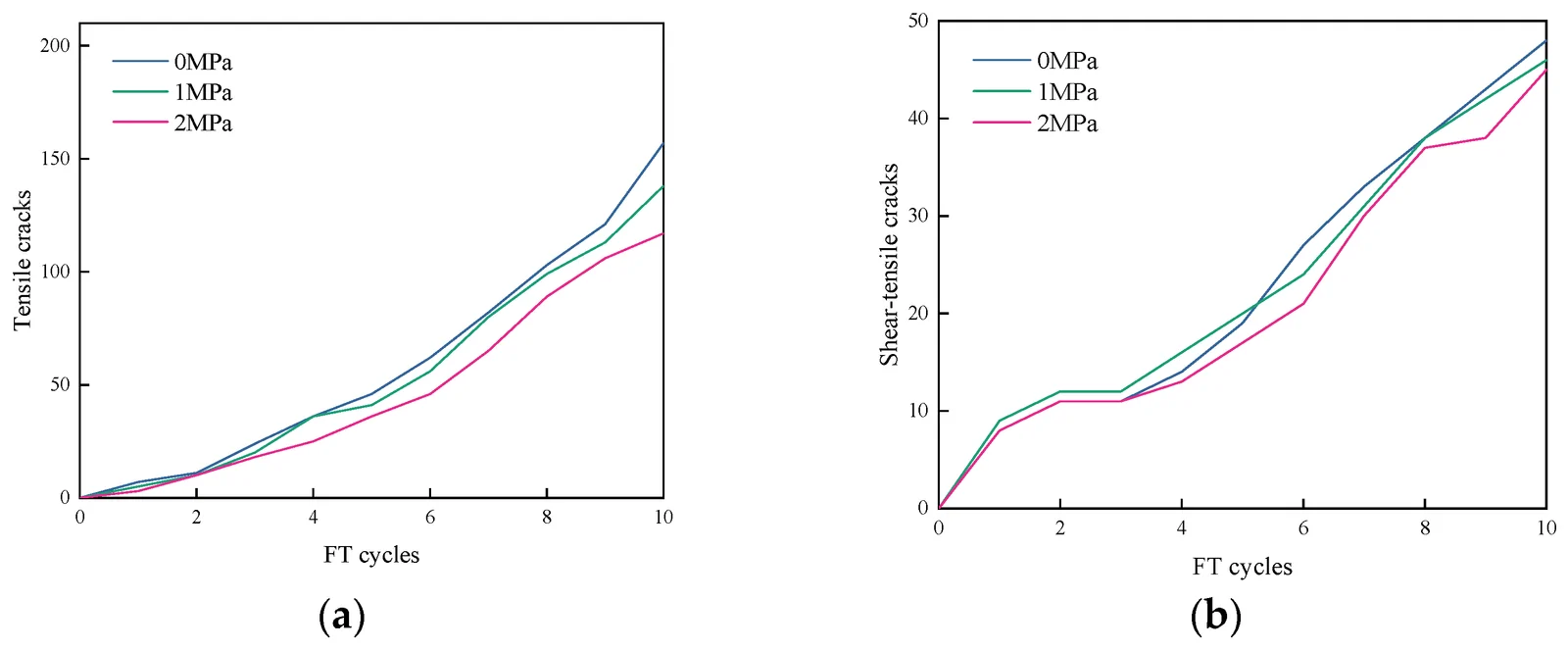
Evolution of the cumulative crack count in the sandstone DEM models under different loading: (**a**) tensile cracks; (**b**) shear–tensile cracks.

To further analyze the contributions of different failure modes to the variation in total crack count, the cumulative numbers of tensile cracks and shear–tensile cracks were counted separately, as shown in Figure 20. The number of tensile cracks continuously increased with the number of freeze–thaw cycles, and its variation trend was generally consistent with that of the total crack count. In the early stage of freeze–thaw cycling, the differences in tensile crack number among different loading groups were small. After six freeze–thaw cycles, the differences between the loaded groups and the no-load group gradually increased. After 10 freeze–thaw cycles, the numbers of tensile cracks in the 0, 1, and 2 MPa groups were 157, 138, and 117, respectively, and those in the 1 MPa and 2 MPa groups were approximately 11.6% and 25.2% lower than that in the no-load group, respectively.

By contrast, shear–tensile cracks showed a relatively rapid growth rate in the early stage of freeze–thaw cycling. After 10 freeze–thaw cycles, the numbers of shear–tensile cracks in the 0, 1, and 2 MPa groups were 48, 46, and 45, respectively, and the differences among the three groups were much smaller than those of tensile cracks. The curves also intersected at some stages, indicating that loading had a weak influence on the absolute number of shear–tensile cracks and did not show a stable promoting or significant inhibitory effect.

In terms of crack composition, tensile cracks accounted for approximately 72–76% of the total crack count after 10 freeze–thaw cycles and were the dominant failure form of freeze–thaw damage. With an increasing loading level, the decrease in tensile crack number was much higher than that in shear–tensile crack number, resulting in a relative increase in the proportion of shear–tensile cracks in the total crack count. However, this proportional change does not mean that sustained loading promoted shear–tensile failure but mainly resulted from the stronger inhibitory effect of sustained axial compressive stress on tensile bond breakage. Therefore, the regulation of freeze–thaw cracks by loading was mainly reflected in limiting the opening deformation between particles and reducing the number of tensile bond failures, while its influence on shear–tensile cracks was relatively limited.

### 5.3. Mechanical Response and Failure Patterns After Load–Freeze–Thaw Treatment

**Figure 21:**
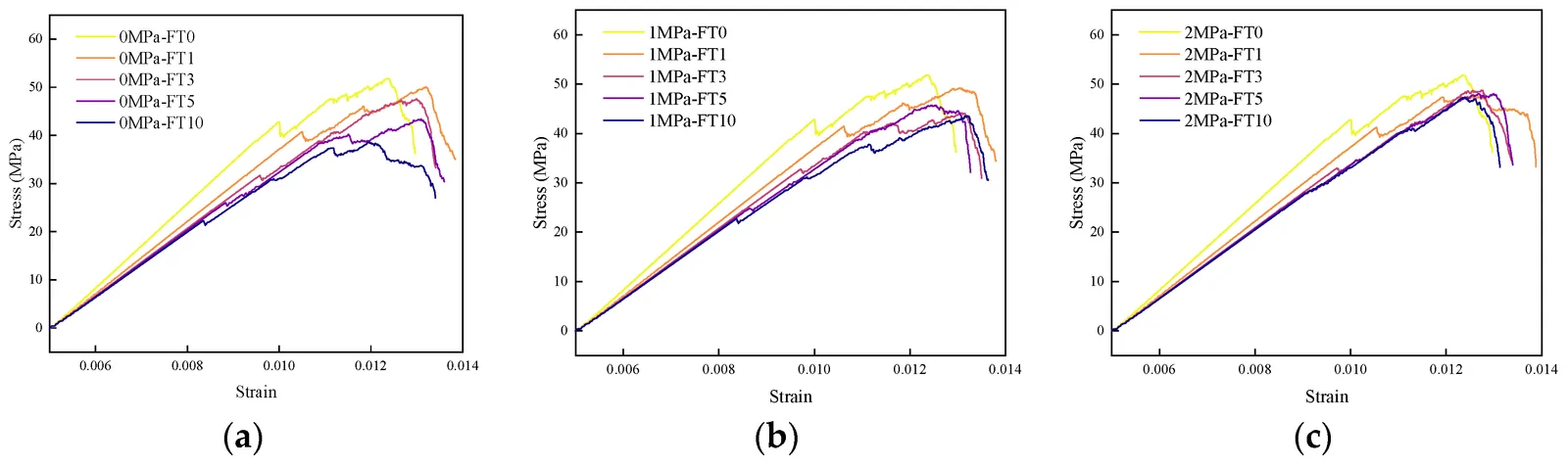
Simulated uniaxial compressive stress–strain curves of sandstone under different load–freeze–thaw conditions: (**a**) 0 MPa; (**b**) 1 MPa; (**c**) 2 MPa.

The simulated uniaxial compressive stress–strain curves of sandstone under different load–freeze–thaw conditions are shown in Figure 21. All curves experienced a nearly linear loading stage, a pre-peak nonlinear deformation stage, and a post-peak decrease in bearing capacity. With increasing freeze–thaw cycles, the peak stress generally decreased, indicating that bond breakage induced by frost heave weakened the subsequent bearing capacity of the model. At the same number of freeze–thaw cycles, the peak stress of the loaded groups was generally higher than that of the no-load group, which was consistent with the overall trend observed in the laboratory tests that loading mitigated strength loss.

The simulated curves of the no-load group reproduced the decreasing trend of compressive strength after freeze–thaw treatment relatively well. For the 1 MPa and 2 MPa loading groups, the model could reflect the basic pattern that the peak stress decreased with increasing freeze–thaw cycles and that the strength retention of the loaded groups was relatively higher. However, the peak variations under different conditions did not form a strict numerical correspondence with the experimental results. The numerical simulation results of loading after load–freeze–thaw treatment were mainly used to evaluate the relative variation trends of mechanical response under different conditions, rather than to provide accurate strength predictions for all loaded conditions.

**Figure 22:**
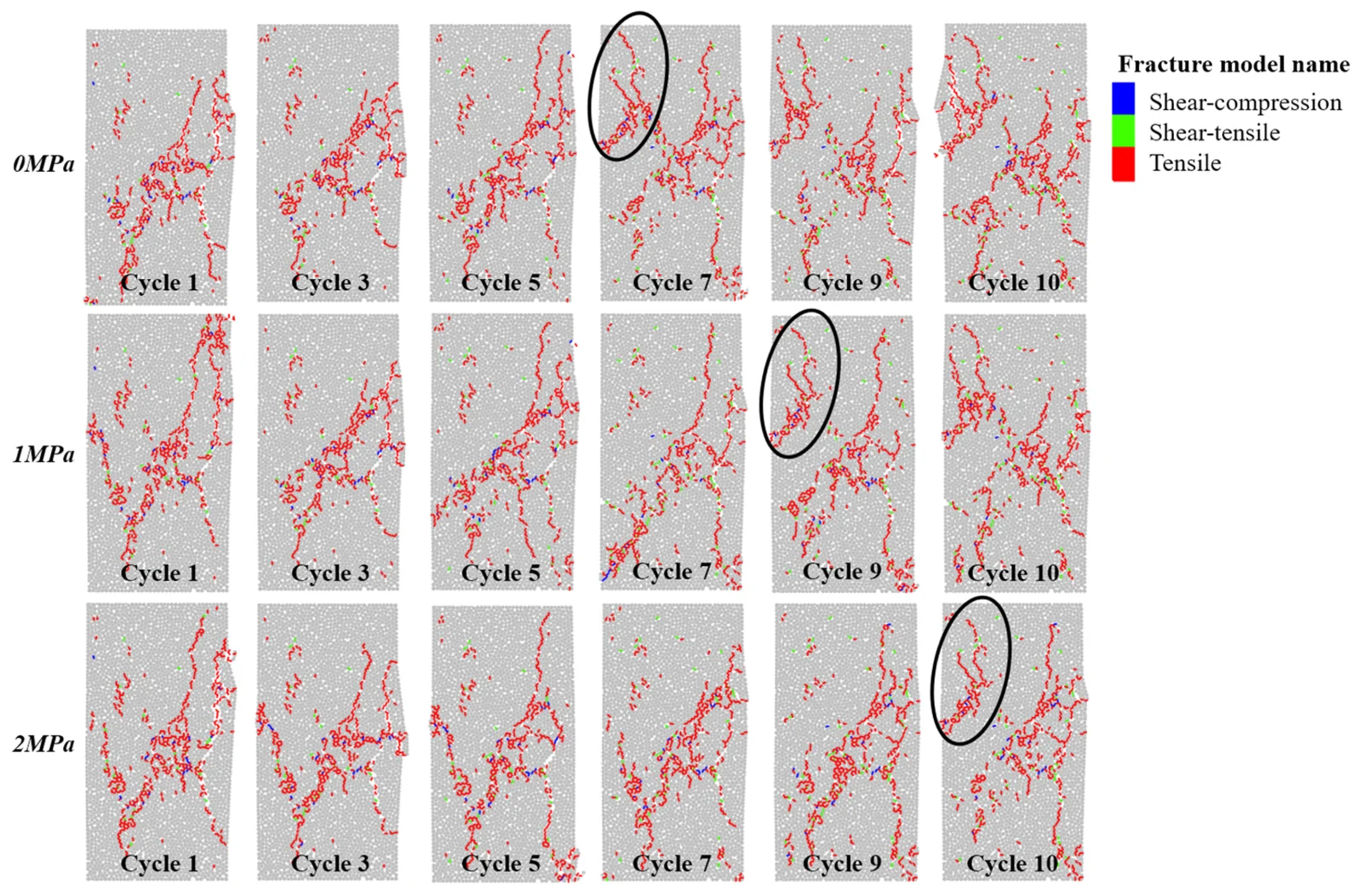
Crack distributions in sandstone models after uniaxial compression under different load–freeze–thaw conditions.

Figure 22 summarizes the crack distributions after uniaxial compression loading under different load–freeze–thaw coupling conditions. After fewer freeze–thaw cycles, the cracks generated by compression loading mainly propagated obliquely through the middle of the specimen and gradually formed a composite failure band dominated by tensile cracks with locally accompanied shear cracks. With increasing freeze–thaw cycles, loading-induced cracks propagated more along pre-existing freeze–thaw cracks and adjacent weak contacts, and the number of branches and spatial range of the main failure band increased accordingly. This indicates that mesoscopic damage formed during the freeze–thaw stage had an obvious guiding effect on the subsequent compression failure path.

The upper crack branch outlined by the black box in Figure 22 was already relatively obvious in the 0 MPa group after seven freeze–thaw cycles, appeared in the 1 MPa group after approximately nine freeze–thaw cycles, and did not form in the 2 MPa group untilten freeze–thaw cycles. This phenomenon indicates that loading reduced the accumulation of tensile cracks during the freeze–thaw stage, delayed local crack connection and secondary branch formation during subsequent compression loading, and was an important reason why the overall strength of the loaded groups was higher than that of the no-load group.

**Figure 23:**
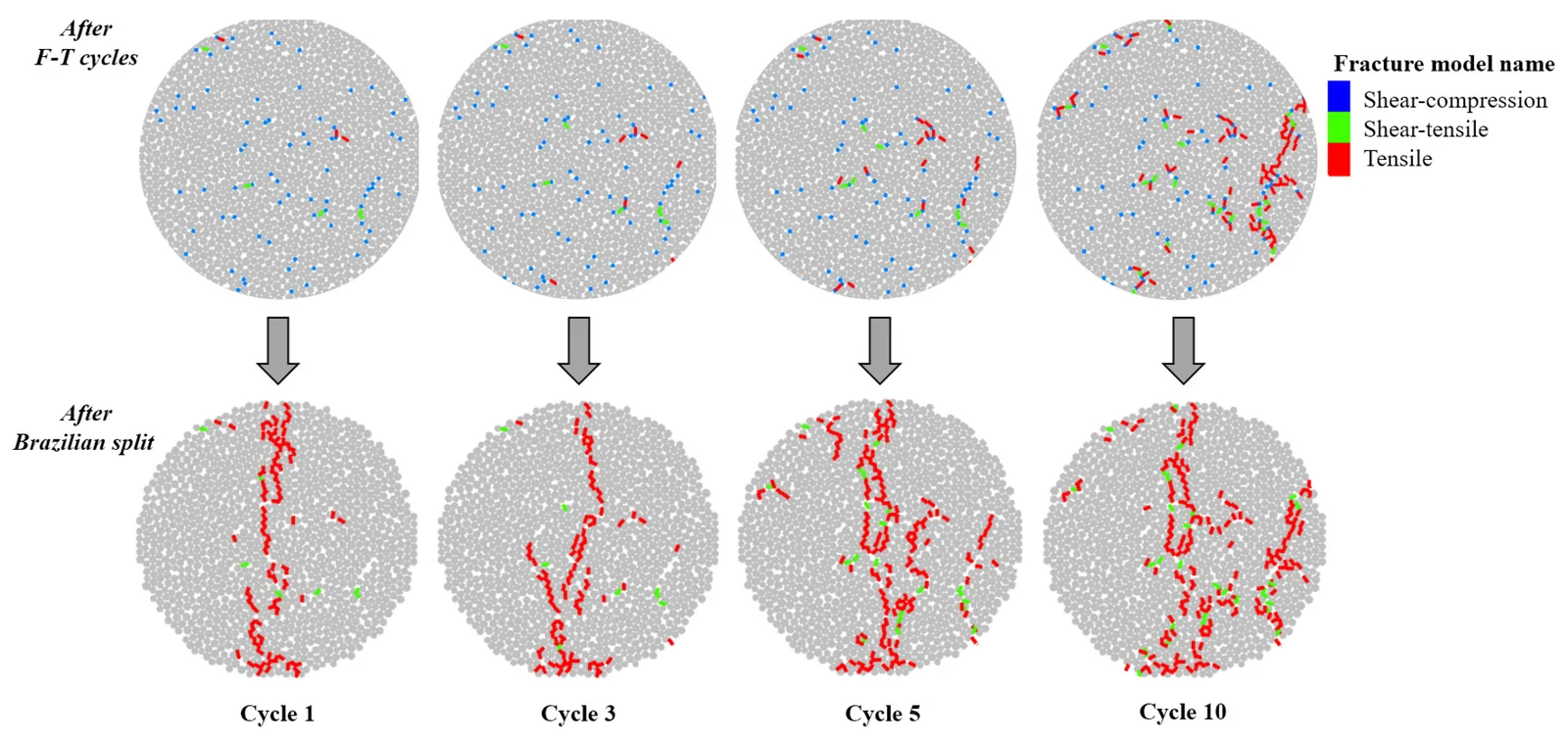
Crack distributions before and after Brazilian splitting at different freeze–thaw cycles under 1 MPa sustained loading.

The pre-existing cracks formed during the freeze–thaw stage not only participated in the formation of subsequent compression failure bands but also affected the propagation and coalescence of the main crack under tensile conditions. Therefore, according to the numerical loading scheme described above, Brazilian splitting loading was carried out on the freeze–thaw-treated disc models. Figure 23 shows the crack distributions before and after loading for models with different numbers of freeze–thaw cycles under 1 MPa sustained loading. The upper row shows the disc models cut from the rectangular parent model after load–freeze–thaw treatment, and the lower row shows the crack distributions of the corresponding specimens after Brazilian splitting loading. With increasing freeze–thaw cycles, the initial damage dominated by tensile cracks gradually accumulated in the discs. In the early stage of freeze–thaw cycling, the main Brazilian splitting crack mainly propagated along the loading diameter, with a relatively continuous path. As damage further developed, the main crack was more likely to connect with adjacent freeze–thaw cracks and produce deflection and branching, gradually widening the through-going failure band. By 10 freeze–thaw cycles, the control effect of pre-existing cracks on the propagation path of the main crack had become very significant. Freeze–thaw cracks provided preferential propagation paths, promoting the connection and coalescence between the main splitting crack and the surrounding damage zones.

### 5.4. Mechanism of Load Influence

The experimental and DEM results jointly indicate that the regulation of sandstone freeze–thaw degradation by loading is not a simple superposition of strength effects. Instead, loading changes the opening and closure states of cracks during freezing, further affecting the accumulation mode of frost-heave damage and the formation of subsequent failure bands. Because the sustained load was removed before the subsequent mechanical tests, the loaded groups still exhibited lower strength degradation rates after unloading, indicating that the strength retention did not result solely from the instantaneous closure of cracks under compression. The DEM results further showed that fewer irreversible parallel-bond break events occurred in the loaded groups during freeze–thaw cycling, particularly fewer tensile cracks. Therefore, sustained loading not only altered the crack opening and closure states but also inhibited the accumulation of irreversible tensile damage during freeze–thaw cycling.

Low-level axial compressive stress exerted a certain closure effect on primary microcracks. During freezing of water-saturated sandstone, the phase transition of pore water produced approximately 9% volumetric expansion. Under the constraint of the particle skeleton and pore walls, frost-heave pressure was generated, promoting the propagation of primary microcracks. After 1–2 MPa sustained axial compressive stress was applied, microcracks intersecting the loading direction at large angles and some oblique cracks tended to close. Within the DEM model, this closure constrained the opening deformation around the equivalent water particles and reduced the tensile bond breakage induced by frost-heave expansion. Sustained loading did not change the basic role of freeze–thaw cycling as the dominant damage factor but changed the evolution rate of frost-heave damage.

This regulatory effect was first reflected in the crack-type composition. With an increasing loading level, the decrease in the total crack count mainly resulted from the reduction in tensile cracks, whereas the absolute number of shear–tensile cracks changed only slightly. This indicates that loading preferentially inhibited opening deformation between particles and tensile bond breakage, resulting in fewer initial defects and weaker connectivity during the freeze–thaw stage, thereby preserving a more complete load-bearing skeleton for subsequent mechanical loading.

Under uniaxial compression, the more pre-existing freeze–thaw cracks there were, the more easily the main failure band branched and extended toward both sides. In the loaded groups, the accumulation of tensile cracks during the freeze–thaw stage was restricted, and the formation of secondary branches was therefore relatively delayed. As a result, the retention effect of loading on compressive strength was more pronounced in the middle stage of freeze–thaw cycling. Under Brazilian splitting, the main crack was more sensitive to internal crack connectivity. As freeze–thaw damage accumulated, pre-existing cracks gradually participated in the coalescence of the main crack and induced deflection and branching. This process was delayed in the loaded groups; so, the inhibitory effect on tensile strength degradation gradually appeared with increasing freeze–thaw cycles. The different responses of the two strength indices to sustained loading essentially reflect the different sensitivities of compressive failure and tensile failure to the initial crack state.

Because the DEM model is two-dimensional, the simulated cracks represent in-plane bond-breakage evolution rather than a complete reconstruction of the three-dimensional fracture network. The present results provide a reference for cold-region rock engineering in which low sustained compressive stress and seasonal freeze–thaw action coexist. Under field conditions, the effects of multiaxial in situ stress, fracture networks, water migration, variable saturation, and nonuniform temperature histories should be further considered in evaluating long-term load–freeze–thaw coupling.

## 6. Conclusions

This study investigated the freeze–thaw degradation of the compressive and tensile properties of water-saturated sandstone under loading through coupled load–freeze–thaw tests and discrete element simulations and analyzed the mechanism by which loading affects freeze–thaw damage. The main conclusions are as follows:

(1) Both the uniaxial compressive strength and Brazilian tensile strength of sandstone decreased with increasing freeze–thaw cycles, but the staged variations of the two strength indices were different. Under the no-load condition, after 10 freeze–thaw cycles, the uniaxial compressive strength and tensile strength decreased by 27.85% and 36.67%, respectively, indicating that the tensile property was more sensitive to freeze–thaw damage.
(2) Sustained axial loading of 1–2 MPa did not change the overall decreasing trend of sandstone strength with increasing freeze–thaw cycles, but it mitigated the strength loss. In general, the inhibitory effect became more evident with increasing loading levels. After 10 freeze–thaw cycles, the uniaxial compressive strength and tensile strength of the 2 MPa group were 9.59% and 10.53% higher than those of the no-load group at the same cycle number, respectively.
(3) Two-variable exponential attenuation models for uniaxial compressive strength and tensile strength were established using the number of freeze–thaw cycles and sustained axial stress as independent variables. In both models, the coefficients of the load-related terms were positive, whereas the no-load attenuation terms were negative. The models can reflect the strength attenuation induced by freeze–thaw cycling and the regulatory effect of sustained loading on the attenuation rate.
(4) Frost-heave cracks in the discrete element simulations continued to accumulate with freeze–thaw cycling. After 10 freeze–thaw cycles, the cumulative total crack counts in the 0, 1, and 2 MPa groups were 205, 183, and 161, respectively, among which tensile cracks accounted for 72–76% of the total crack count. The reduction in total crack count caused by the increasing loading level mainly resulted from the decrease in tensile cracks, whereas the absolute number of shear–tensile cracks changed only slightly.
(5) During freeze–thaw cycling, loading mainly closed microcracks intersecting the loading direction at large angles and some oblique cracks, limiting the opening deformation between particles and tensile bond breakage, thereby slowing the propagation and local coalescence of frost-heave cracks.

## Figures and Tables

**Table 1:** Test scheme for load–freeze–thaw coupling treatment.

Group	Axial Stress (MPa)	Number of Freeze–Thaw Cycles
Control group	0	0
Single freeze–thaw group	0	1, 3, 5, 10
Load–freeze–thaw coupled group I	1	1, 3, 5, 10
Load–freeze–thaw coupled group II	2	1, 3, 5, 10

**Table 2:** Comparison between fitted values and measured mean values, together with the corresponding error statistics.

Number of Freeze–Thaw Cycles, N	Sustained Axial Stress /MPa	Measured Compressive Strength /MPa	Fitted Compressive Strength /MPa	Relative Error /%	Measured Tensile Strength /MPa	Fitted Tensile Strength /MPa	Relative Error /%
0	0	55.721	55.721	0	3.30	3.30	0
1	0	51.872	53.531	3.2	3.18	3.15	0.9
	1	52.300	53.960	3.2	3.18	3.17	0.3
	2	53.552	54.394	1.6	3.24	3.19	1.5
3	0	48.267	49.405	2.4	2.81	2.87	2.1
	1	50.223	50.605	0.8	2.87	2.93	2.1
	2	51.689	51.833	0.3	2.93	2.98	1.7
5	0	41.730	45.598	9.2	2.63	2.62	0.4
	1	45.335	47.457	4.5	2.69	2.70	0.4
	2	48.940	49.393	0.9	2.87	2.79	2.8
10	0	40.202	37.314	7.2	2.09	2.08	0.5
	1	42.341	40.419	4.5	2.25	2.21	1.8
	2	44.058	43.784	0.6	2.31	2.35	1.7

**Table 3:** Calibrated micro-parameters of the DEM model.

Particle	Rock	Water	Bond	
Minimum radius (mm)	0.58	0.38	Elastic modulus (GPa)	4.12
Maximum radius (mm)	0.78	0.38	Stiffness ratio	1.83
Density (kg/m^2^)	2400	1000	Tensile strength (MPa)	50.13
Friction coefficient	0.17	0.01	Cohesion (MPa)	43.91

## Data Availability

The original contributions presented in this study are included in the article. Further inquiries can be directed to the corresponding author.
